# Biochemical and cellular characterisation of the *Plasmodium falciparum* M1 alanyl aminopeptidase (*Pf*M1AAP) and M17 leucyl aminopeptidase (*Pf*M17LAP)

**DOI:** 10.1038/s41598-021-82499-4

**Published:** 2021-02-03

**Authors:** Rency Mathew, Juliane Wunderlich, Karine Thivierge, Krystyna Cwiklinski, Claire Dumont, Leann Tilley, Petra Rohrbach, John P. Dalton

**Affiliations:** 1 Institute of Parasitology, McGill University, 21111 Lakeshore Road, Sainte-Anne-de-Bellevue, Québec, H9X 3V9 Canada; 2 School of Biological Sciences, Queen’s University Belfast, Belfast, Northern Ireland UK; 3 European Molecular Biology Laboratory, Notkestraße 85, 22607 Hamburg, Germany; 4 Laboratoire de Santé Publique du Québec, Institut National de Santé Publique du Québec, Sainte-Anne-de-Bellevue, QC Canada; 5 Centre for One Health & Ryan Institute, School of Natural Sciences, NUI Galway, Galway, Republic of Ireland; 6 Department of Biochemistry and Molecular Biology, Bio21 Institute, University of Melbourne, Melbourne, VIC Australia

**Keywords:** Biochemistry, Cell biology

## Abstract

The *Plasmodium falciparum* M1 alanyl aminopeptidase and M17 leucyl aminopeptidase, *Pf*M1AAP and *Pf*M17LAP, are potential targets for novel anti-malarial drug development. Inhibitors of these aminopeptidases have been shown to kill malaria parasites in culture and reduce parasite growth in murine models. The two enzymes may function in the terminal stages of haemoglobin digestion, providing free amino acids for protein synthesis by the rapidly growing intra-erythrocytic parasites. Here we have performed a comparative cellular and biochemical characterisation of the two enzymes. Cell fractionation and immunolocalisation studies reveal that both enzymes are associated with the soluble cytosolic fraction of the parasite, with no evidence that they are present within other compartments, such as the digestive vacuole (DV). Enzyme kinetic studies show that the optimal pH of both enzymes is in the neutral range (pH 7.0–8.0), although *Pf*M1AAP also possesses some activity (< 20%) at the lower pH range of 5.0–5.5. The data supports the proposal that *Pf*M1AAP and *Pf*M17LAP function in the cytoplasm of the parasite, likely in the degradation of haemoglobin-derived peptides generated in the DV and transported to the cytosol.

## Introduction

*Plasmodium falciparum* is the causative agent of the most lethal form of malaria. In 2018, there was an estimated 228 million cases of malaria that resulted in ~ 405,000 deaths, predominantly in Africa and consisting mainly of children and pregnant women^1^. The global spread of drug-resistant parasites has left few effective treatments, the most important of which is artemisinin and its derivatives. Artemisinin combination therapy-resistant malaria cases have been reported in South-East Asia, causing alarm and highlighting the need for new antimalarial agents with alternate modes of action^2^.

Malaria parasites display abundant intracellular aminopeptidase activity with optimal activity in the neutral pH range^3–5^. Neutral aminopeptidase activity was suggested to function in the terminal stages of protein degradation in the parasite cytoplasm^4,6,7^. Since its activity exhibited a distinct preference for synthetic substrates containing N-terminal leucine or alanine, residues that are most abundant in haemoglobin (24%), a role in the catabolism of this red cell protein was implied. Thus, it was proposed that peptides, derived from the action of various proteolytic peptidases on haemoglobin in the specialised acidic digestive vacuole (DV), were transported to the cytoplasm. Here, neutral aminopeptidases were thought to process these peptides to free amino acids that are then used in parasite protein synthesis^4,8,9^.

Only two single-copy genes encoding neutral aminopeptidases are present in the *P. falciparum* 22.9-Mb genome. Their structure and classification are different, as are their substrate preference and mechanism of cleavage. Florent et al.^10^ described a monomeric M1-family alanyl aminopeptidase (*Pf*M1AAP) with a trans-membrane domain within an extended N-terminal region. Allary et al.^11^ showed that *Pf*M1AAP is expressed as a 120-kDa protein that is proteolytically processed to a fragment of 96 kDa lacking the N-terminal trans-membrane domain, and a 68-kDa fragment that results from additional cleavage at the C-terminal end. The second neutral aminopeptidase is a 67.8-kDa M17-family leucyl aminopeptidase (*Pf*M17LAP) with no trans-membrane region^6^. The native aminopeptidase is a homo-hexameric enzyme, with each subunit detected as a single 68–70-kDa protein in SDS-PAGE analysis of soluble freeze–thaw extracts of *P. falciparum* malaria parasites^9,12^.

Biochemical studies showed that *Pf*M1AAP and *Pf*M17LAP are metallo-exopeptidases with overlapping but distinct substrate specificity profiles^7,13^. *Pf*M1AAP efficiently hydrolyses N-terminal leucine, alanine, arginine, and lysine, and cleaves phenylalanine, tyrosine, serine, and asparagine at lower catalytic rates. In contrast, *Pf*M17LAP exhibits an almost exclusive specificity for leucine and tryptophan^6,7,10,11^; studies showed that hydrolysis of N-terminal alanine, proline or phenylalanine is poor, and the enzyme is unable to cleave N-terminal valine, glycine, asparagine, glutamine, isoleucine or arginine^5,6^. Attempts to knockout the *Pf*M1AAP and *Pf*M17LAP genes were not successful suggesting the absence of functional redundancy between the two enzymes and that both are essential for parasite survival within the erythrocyte^6,7,14^.

Both aminopeptidases are of prime interest because they are validated targets at which new anti-malarial drugs could be directed. Several aminopeptidase inhibitors with broad specificity, such as bestatin and compound 4, kill *P. falciparum* parasites in vitro, while a hydroxamate-containing compound CHR-2863 was shown to inhibit the growth of murine malaria *P. chabaudi chabaudi *in vivo^5,9,15–17^. Recent efforts in anti-aminopeptidase drug design has been focused on the development of inhibitors that are specific to malaria aminopeptidases since homologs are found in the human host^18–20^. To increase potency, dual inhibitors of *Pf*M1AAP and *Pf*M17LAP are also being explored^21–23^. Despite differences between the active site of the two enzymes, sufficient similarities within the S1 and S1′ pockets have allowed the development of novel analogues of hydroxamic acid with dual enzyme inhibitory action and the ability to block *P. falciparum* growth in vitro at IC50 of ~ 96 nM^24^.

In the pursuit of anti-malaria drugs directed at aminopeptidases, aspects of the basic biochemistry and cellular biology of these pivotal enzymes were neglected. This information will be useful to understand the action of inhibitory compounds, especially dual-pronged compounds, and facilitate their future optimisation. Earlier studies have often focused on one or other enzyme, and discrepancies regarding cellular location and putative function(s) have arisen. In the present study, we have performed comparative biochemical, cell fractionation, and immunolocalisation studies on both *Pf*M1AAP and *Pf*M17LAP that add new insights to their putative intra-cellular location and function. In conformity with Allary et al.^11^, we show that *Pf*M1AAP is proteolytically cleaved in the parasite cytoplasm to give rise to a ~ 100-kDa and ~ 70-kDa fragment, the latter of which lacks a C-terminal domain that is essential for functionality.

## Results

### Distribution of *Pf*M1AAP and *Pf*M17LAP in different malaria parasite fractions

**Figure 1:**
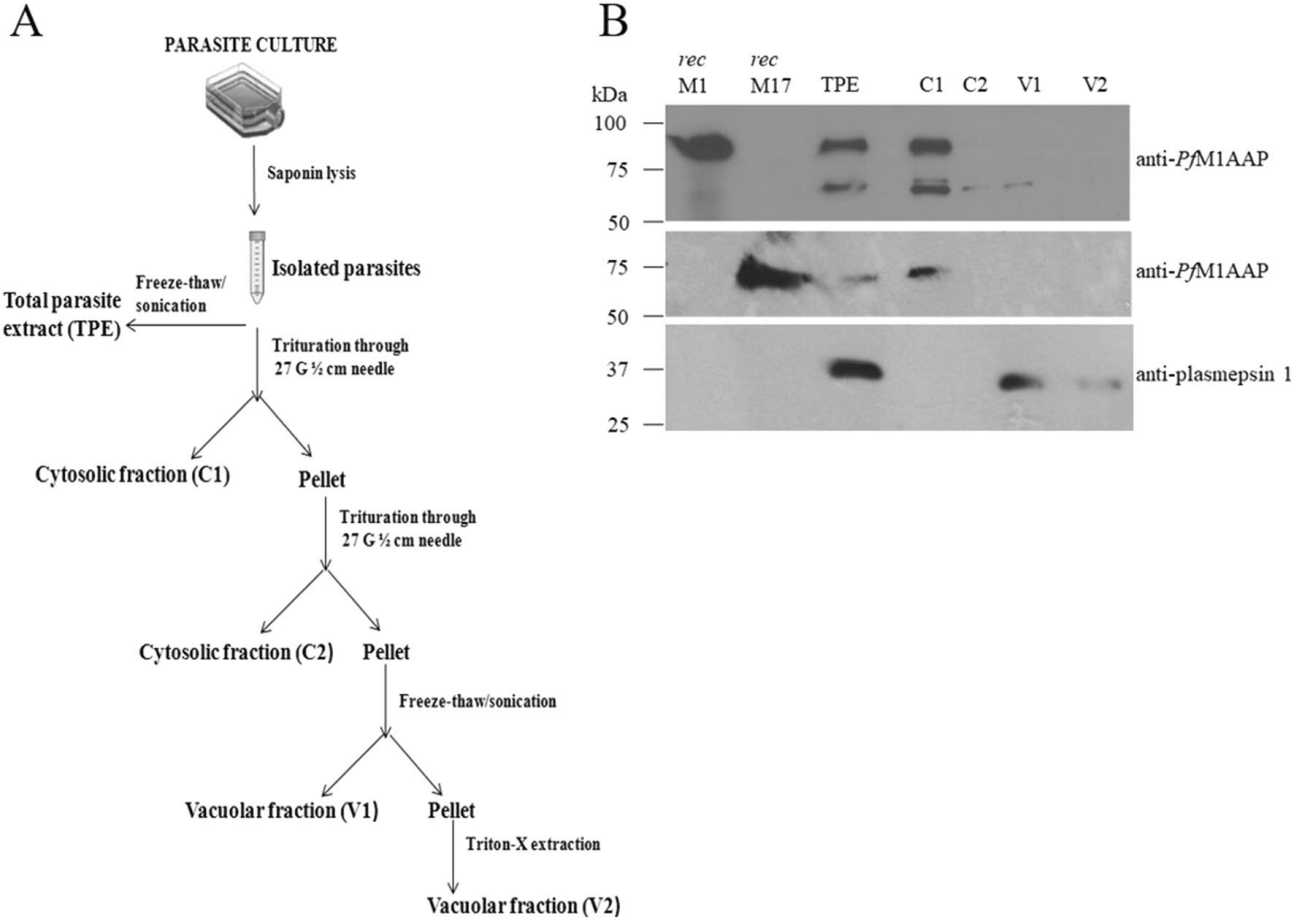
Distribution of *Pf*M1AAP and *Pf*M17LAP in the *P. falciparum* cellular compartments. (**A**) Flowchart depicting the isolation of *P. falciparum* parasites from host erythrocytes followed by fractionation of cell compartments. Parasites were isolated by saponin lysis of erythrocytes. Total parasite extracts (TPE) were prepared by freeze–thaw and sonication of the parasites in 10 mM Tris–HCl buffer, pH 7.2. Other samples were triturated four times through a syringe needle and centrifuged to obtain the first cytosolic fraction, C1, and a pellet. The pellet was resuspended in 10 mM Tris–HCl buffer, pH 7.2 and triturated/centrifuged to obtain the second cytosolic fraction, C2, and a pellet. This pellet was re-suspended in 10 mM Tris–HCl buffer, pH 7.2, and subjected to four rounds of freeze–thaw treatment followed by centrifugation to obtain a soluble vacuolar fraction, V1, and a pellet. The final detergent-soluble vacuolar fraction, V2, was obtained by incubating the pellet in 0.5% Triton X for 30 min on ice. (**B**) Representative immunoblots of three biological replicates showing the *P. falciparum* recombinant (rec) *Pf*M1AAP, rec *Pf*M17LAP, total parasite extract (TPE) and cellular fractions (C1, C2, V1 and V2) probed with anti-*Pf*M1AAP (top panel), anti-*Pf*M17LAP (middle panel), and anti-plasmepsin 1 antibodies (lower panel). Chemiluminescent molecular weight standards are shown on the left.

To examine the distribution of *Pf*M1AAP (PF3D7_1311800) and *Pf*M17LAP (PF3D7_1446200) in blood stage *P. falciparum*, we prepared subcellular fractions using modifications from the protocol of Saliba et al.^25^. Parasites released from erythrocytes using saponin detergent where rapidly lysed by trituration in a hypotonic solution at pH 7.2. The lysed parasites were separated into a soluble fraction, representing the parasite cytosol (C1 and C2), and a non-soluble fraction containing DVs and other organelles (V1 and V2) (see flowchart, Fig. 1A). These fractions were then subjected to immunoblotting and probed with antibodies prepared against recombinant *Pf*M1AAP and *Pf*M17LAP.

Immunoblots of total parasite extract (TPE) probed with anti-*Pf*M1AAP antibodies detected a 100-kDa protein, that co-migrated with recombinant *Pf*M1AAP, and a second protein of about ~ 70 kDa (Fig. 1B; Supplementary Fig. 9). These two bands were also detected in the first cytosolic fraction (C1). A minor fraction of the 70-kDa protein was observed in second cytosolic fraction (C2) and the V1 fraction, which is a freeze–thaw extract of the vacuolar fraction. None of these three proteins were found in the final Triton-X-extracted vacuolar fraction (V2) (Fig. 1B).

A single protein that migrated close to the 60-kDa molecular size marker and co-migrated with the 58-kDa recombinant *Pf*M17LAP was detected in TPE using anti-*Pf*M17LAP. This protein was only detected in the first C1 fraction, a distribution expected for a solely cytoplasmic enzyme (Fig. 1B).

The immunoblots probed with anti-plasmepsin I detected a protein close to the molecular size standard of 40 kDa in TPE and in the freeze–thaw extract of the vacuolar fraction, V1 but not in the cyoplasmic fractions (Fig. 1B). This is consistent with Banerjee et al.^26^, where it is reported that plasmepsin I locates to the DV and is processed to a 37-kDa mature enzyme from a 51-kDa proenzyme.

To quantify the relative amount of *Pf*M1AAP in each cellular fraction, we calculated the total enzymatic units in each fraction using the fluorogenic peptide substrate H-Arg-NHMec, which is specific for this aminopeptidase^6,7,27^ (Table 1). We also measured the enzymatic units in the fraction with H-Leu-NHMec, which is cleaved by both *Pf*M1AAP and *Pf*M17LAP^27^. Approximately, 85% *Pf*M1AAP activity was associated with the cytosolic fractions (C1 and C2), while about 10% was found in the freeze–thaw extracts of the vacuolar fraction (V1) with the residual activity in the detergent extract of the vacuolar fraction (V2). Activity measured using H-Leu-NHMec activity was highest in the cytosolic fractions C1 and C2 (total ~ 90%) with less than 10% associated with the vacuolar fractions (V1 and V2) (Table 1).

**Table 1:** Relative distribution of aminopeptidase activity in the *P. falciparum* cellular fractions (C1, C2, V1 and V2) calculated from a standard curve prepared using free NMMec.

Fraction	H-Arg-NHMec		H-Leu-NHMec	
	Enzyme activity (Units^a^)	Relative activity (%)	Enzyme activity (Units^a^)	Relative activity (%)
Total	178 ± 15.6	100	133.6 ± 1.1	100
Cytosolic 1 (C1)	145.5 ± 19.6	80 ± 7.5	116.4 ± 17.3	85 ± 10.5
Cytosolic 2 (C2)	13 ± 9.3	6.8 ± 4.3	8.4 ± 7.9	5.8 ± 4.9
Vacuolar 1 (V1)	17.5 ± 12.6	9.4 ± 6.7	11.3 ± 8.7	8.3 ± 6.9
Vacuolar 2 (V2)	6.4 ± 5.2	3.8 ± 3.3	1.3 ± 1.5	1.1 ± 1.2

^a^One unit of enzyme is defined as the nanomoles of NHMec released per 30 min at 37 °C.

### Protease-mediated degradation of *Pf*M1AAP

**Figure 2:**
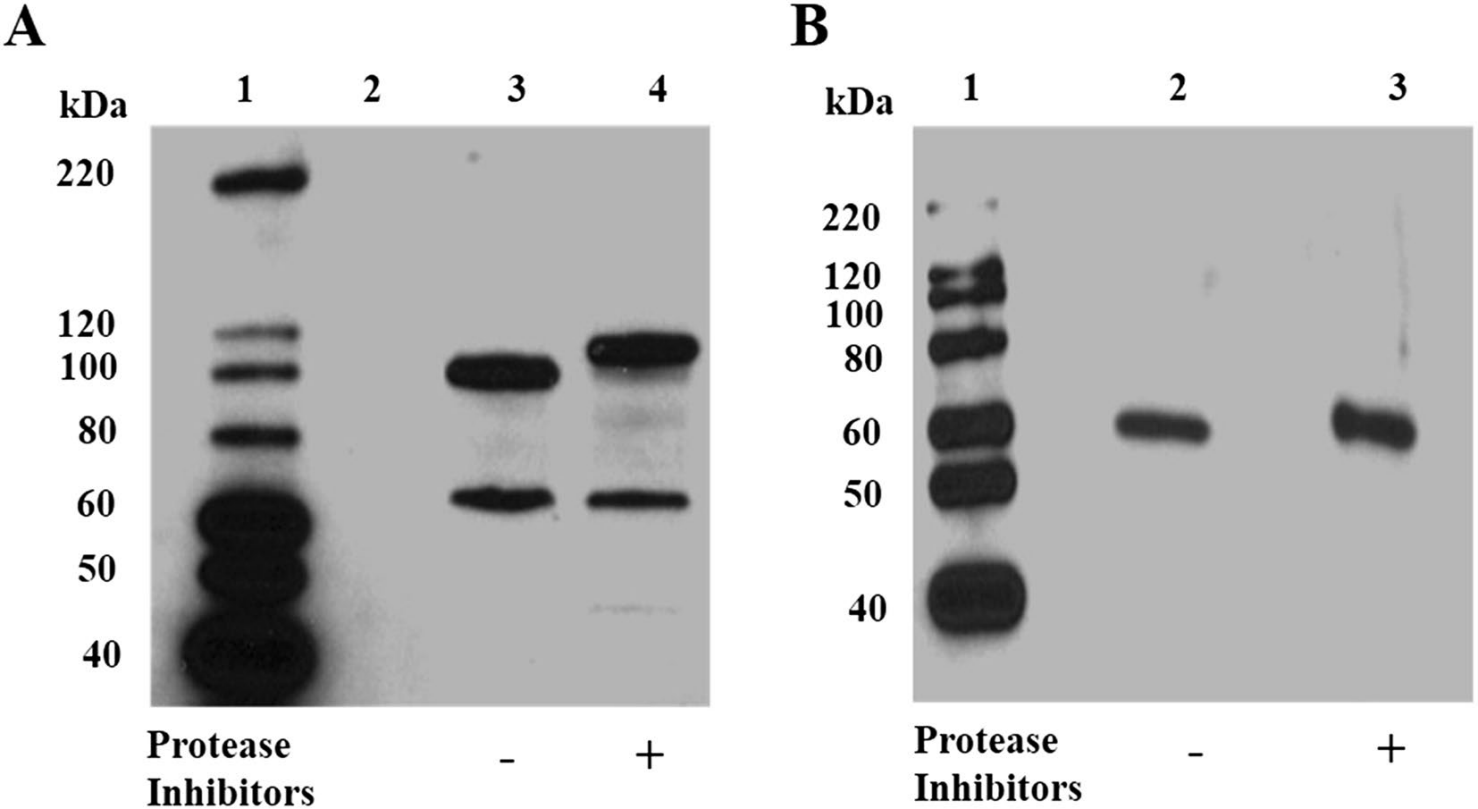
Detection of native *Pf*M1AAP and *Pf*M17LAP in malaria cytosolic extracts. Immunoblots of cytosolic fractions (C1) prepared either without (−) or with (+) a cocktail of protease inhibitors were probed with (**A**) anti-*Pf*M1AAP, and (**B**) anti-*Pf*M17LAP polyclonal antibodies prepared in rabbits and adsorbed against *E. coli* extracts (see “Materials and methods”). The chemiluminescent molecular size markers are shown on the left of each blot (lane 1).

The cellular fractionation studies described above were intentionally prepared in the absence of protease inhibitors. To determine if protease cleavage is occurring, we prepared cytosolic fractions using trituration in hypotonic solution, pH 7.2, with and without a cocktail of protease inhibitors and analysed these by SDS-PAGE followed by immunoblotting. In the absence of protease inhibitors, the *Pf*M1AAP migrates as two bands at approximately 70 kDa and 100 kDa (Fig. 2A, lane 3). When protease inhibitors were added to the preparation, *Pf*M1AAP migrates at approximately 70 kDa and 115 kDa (Fig. 2A, lane 4). Immunoblotting analysis of the same extracts probed with anti-*Pf*M17LAP detected only the protein at ~ 60 kDa in both fractions (Fig. 2B, lanes 2 & 3).

**Figure 3:**
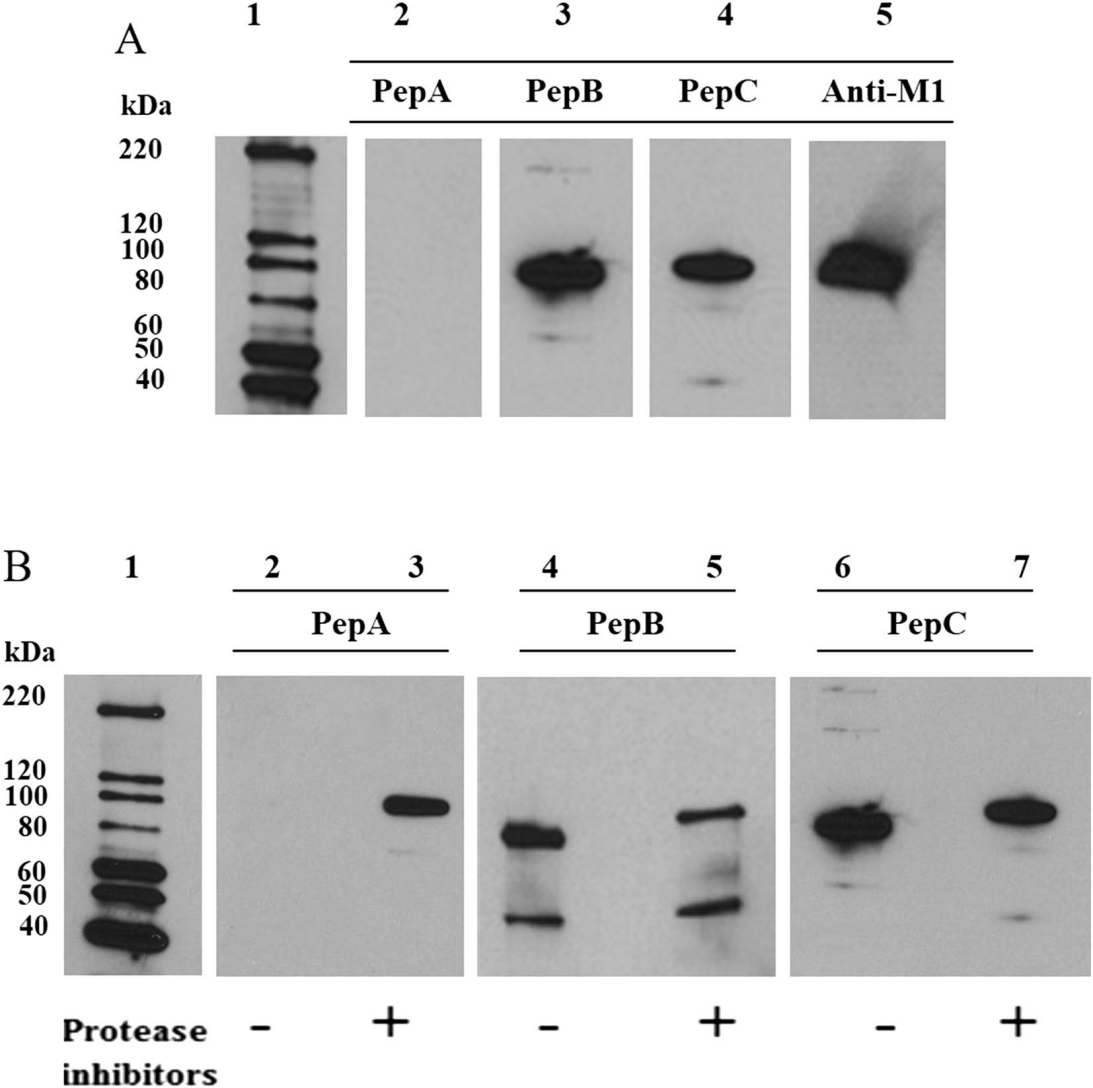
Immunoblots with peptide antibodies. (**A**) Recombinant *Pf*M1AAP was probed with antibodies prepared against 14-mer peptides derived from the N-terminal extension (PepA), spanning domain 1 (PepB) and domain 4 (PepC) (see Supplementary Fig. 1). Anti-*Pf*M1AAP was used as a positive control. Anti-PepA does not react with r*Pf*M1AAP, as this lacks the N-terminal extention, whereas both anti-PepB and anti-PepC do. Lane 1 was loaded with chemilluminescent molecular marker. Lanes 2–5 were loaded with 0.25 µg of recombinant M1. (**B**) Cytosolic fractions of malaria parasites prepared without (−) and with (+) a protease inhibitor cocktail were probed with anti-PepA, anti-PepB and anti-PepC antibodies. Extracts were separated on 4–15% SDS-PAGE gels and transferred to nitrocellulose membrane. Lanes 2 and 3 were probed with anti-PepA, lanes 4 and 5 with anti-PepB and lanes 6 and 7 with anti-PepC antibodies. The chemiluminescent molecular marker is shown in lane 1.

We prepared antibodies against three different peptides sequences within *Pf*M1AAP and used these to probe cytosolic extracts prepared in the presence and absence of protease inhibitors (Fig. 3A; Supplementary Figs. 1, 9). One of these antibodies, anti-PepA, was prepared against a sequence located within the N-terminal extension and thus did not react with our recombinant *Pf*M1AAP that lacked this region. Interestingly, anti-PepA detected the 115-kDa *Pf*M1AAP protein within the cytosolic extracts prepared in the presence of protease inhibitors but did not react with any proteins in extracts prepared in the absence of inhibitors (Fig. 3B, lanes 2 and 3; Supplementary Fig. 9), indicating that the N-terminal extension of the 115-kDa protein is proteolytically cleaved during the extraction process. Anti-PepB, which was prepared against a peptide derived from domain 1 of *Pf*M1AAP, detected the 70-kDa and the 115-kDa protein in cytosolic extracts prepared in the presence of inhibitors and the 70-kDa and 100-kDa proteins in extracts prepared without inhibitors (Fig. 3B, lanes 4 and 5). Finally, anti-PepC, which was prepared against a sequence within the C-terminal domain 4 of *Pf*M1AAP, detected the 115-kDa protein in cytosolic extracts prepared in the presence of inhibitors and the 100-kDa protein in extracts prepared without inhibitors but did not detect the 70-kDa component (Fig. 3B, lanes 6 and 7).

Finally, we incubated recombinant *Pf*M1AAP with the parasite cytosolic fractions that were extracted with and without protease inhibitors overnight at 37 °C to examine whether this was susceptible to cleavage by proteases. Immunoblots show a strong single protein at 100 kDa corresponding to the recombinant enzyme and no evidence of degradation products (Supplementary Fig. 2).

### Localization of native *Pf*M1AAP and *Pf*M17LAP in intra-erythrocytic *P. falciparum* 3D7 parasites

**Figure 4:**
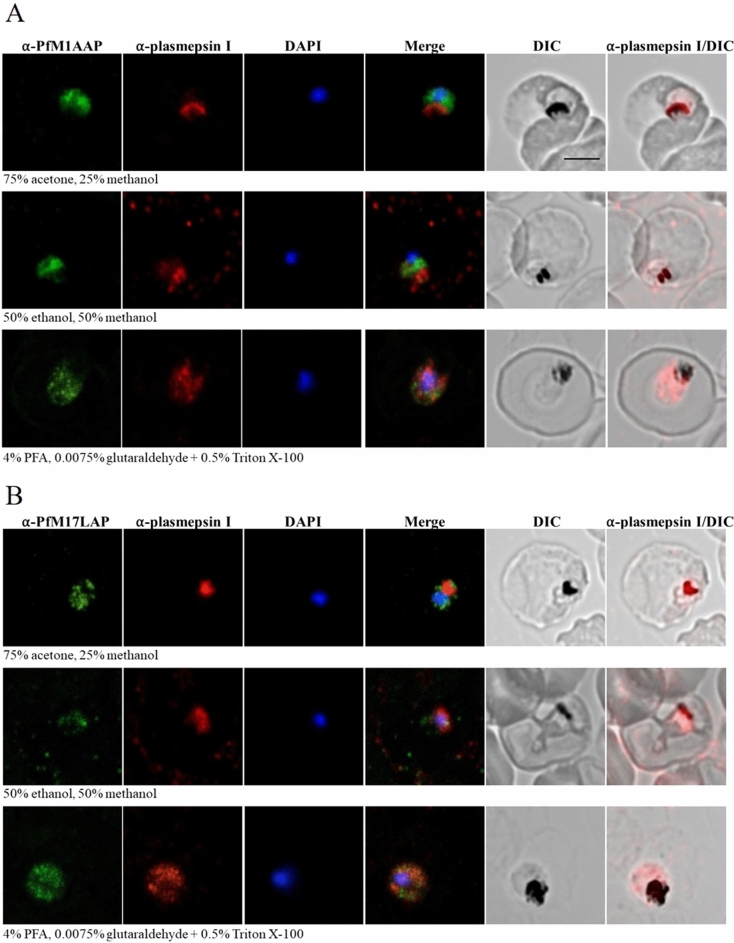
Localization of *Pf*M1AAP and *Pf*M17LAP in intra-erythrocytic *P. falciparum* 3D7 trophozoite-stage parasites. Immunofluorescence assays were carried out using air-dried blood smears fixed with 75% acetone and 25% methanol at − 20 °C for 5 min, or 50% ethanol and 50% methanol at − 20 °C for 2 min, or 4% PFA and 0.0075% glutaraldehyde for 20 min at room temperature. Fixed parasites were probed with polyclonal antibodies against (**A**) *Pf*M1AAP and (**B**) *Pf*M17LAP. Specific aminopeptidase staining (green, Alexa-Fluor 488) was observed in the cytosol of parasites. Parasite nuclei were visualized using DAPI (blue; 4,6-diamidino-2-phenylindole) and monoclonal antibodies against the DV marker plasmepsin I (α-plasmepsin I, red, Alexa-Fluor 594) were used as a control. Differential interference contrast (DIC) and α-plasmepsin I with DIC are shown for reference. Scale bar, 3 µm.

**Figure 5:**
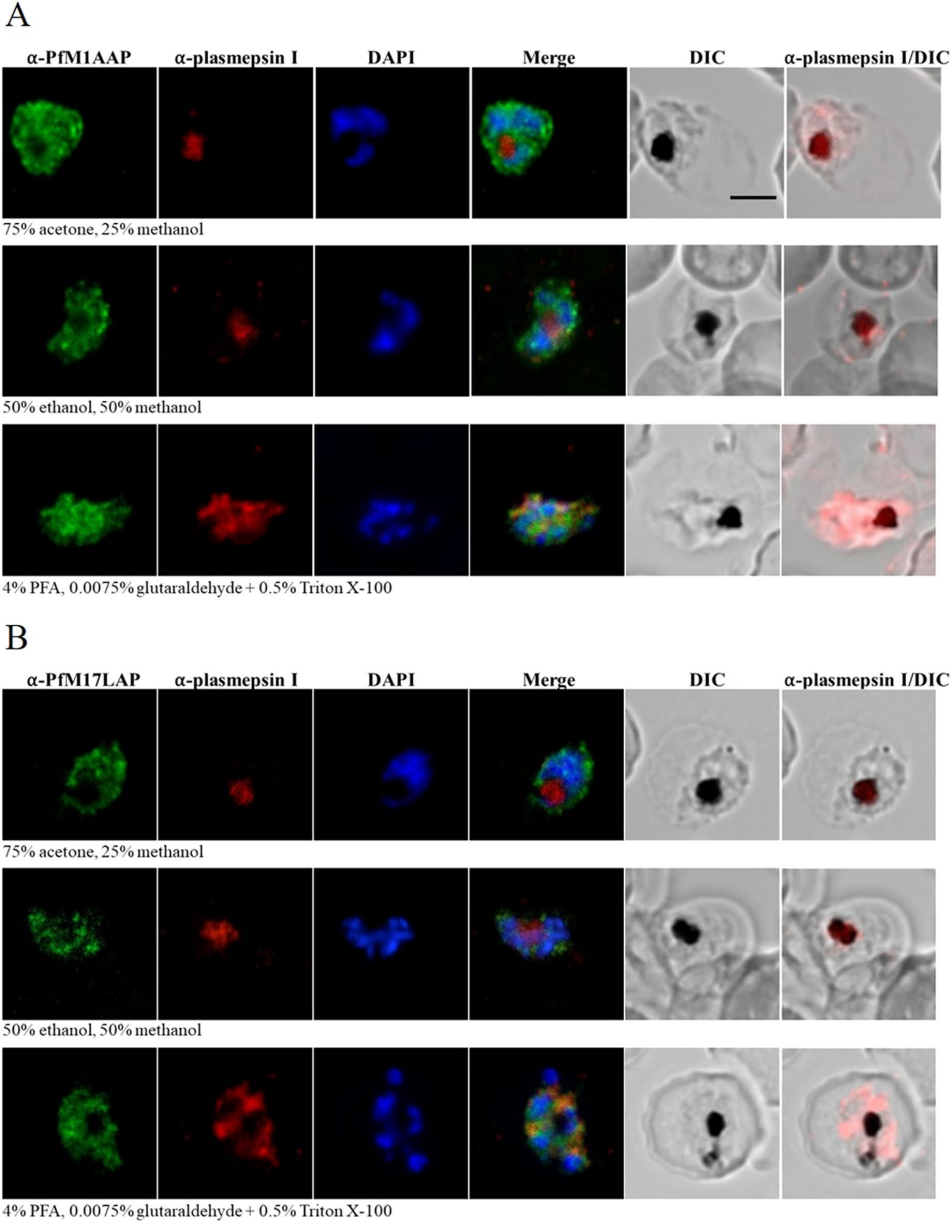
Localization of *Pf*M1AAP and *Pf*M17LAP in intra-erythrocytic *P. falciparum* 3D7 schizont-stage parasites. Immunofluorescence assays were carried out using air-dried blood smears fixed with 75% acetone and 25% methanol at − 20 °C for 5 min or 50% ethanol and 50% methanol at − 20 °C for 2 min or 4% PFA and 0.0075% glutaraldehyde for 20 min at room temperature. Fixed parasites were probed with polyclonal antibodies against (**A**) *Pf*M1AAP and (**B**) *Pf*M17LAP. Specific aminopeptidase staining (green, Alexa-Fluor 488) was observed in the cytosol of parasites. Parasite nuclei were visualized using DAPI (blue; 4,6-diamidino-2-phenylindole) and monoclonal antibodies against the DV marker plasmepsin I (α-plasmepsin I, red, Alexa-Fluor 594) were used as a control. Differential interference contrast (DIC) and α-plasmepsin I with DIC are shown for reference. Scale bar, 3 µm.

To determine the intracellular localization of endogenous *Pf*M1AAP and *Pf*M17LAP in parasite-infected erythrocytes, immunofluorescence assays were carried out on various intra-erythrocytic stages and visualized by confocal microscopy (Figs. 4 and 5). Blood smears of *P. falciparum-*infected erythrocytes were air-dried and fixed using three different methods: (a) parasites were fixed and permeabilized with a mixture of 75% acetone and 25% methanol for 5 min at − 20 °C^10,11,28^ (b) parasites were fixed and permeabilized in a mixture of 50% ethanol and 50% methanol for 2 min at − 20 °C^29^ and (c) parasites were fixed by incubating with 4% paraformaldehyde (PFA) and 0.0075% glutaraldehyde for 20 min at room temperature and treated with 0.5% Triton X-100 for 10 min for permeabilization^14,29,30^. Fixed parasites were probed with polyclonal antibodies against *Pf*M1AAP and *Pf*M17LAP that were adsorbed against *E. coli* to remove antibodies that may bind non-specifically. Using Alexa-Fluor 488-conjugated secondary antibodies, fluorescence was only seen in parasitized erythrocytes, as confirmed by DAPI fluorescence arising from the parasite nuclei. Monoclonal antibodies against plasmepsin I were used as a control for DV localization.

For both anti-*Pf*M1AAP and anti-*Pf*M17LAP antibodies and for all fixation methods used, a diffuse cytosolic fluorescence pattern containing some cytoplasmic spots excluded from the nucleus (as determined by calculating the Pearson’s correlation coefficient as described in^31^) and the DV lumen was observed in trophozoite-stage parasites (Fig. 4). Schizont-stage parasites showed more fluorescence, which was observed as patches in the cytoplasm (Fig. 5). As expected, anti-plasmepsin I antibody fluorescence was found in the DV lumen and co-localised with hemozoin in samples fixed with acetone/methanol or ethanol/methanol. Some cytosolic fluorescence was detected with anti-plasmepsin I antibodies in parasites fixed with PFA and glutaraldehyde and permeabilized with Triton X-100 at 0.5%, which may indicate that this procedure causes redistribution of plasmepsin I in the cytosol and DV (Figs. 4 and 5^32^).

Further immunolocalisation studies were performed in a separate laboratory (L. Tilley, University of Melbourne, Australia) using paraformaldehyde/glutaraldehyde and acetone-fixed parasites and concluded that both *Pf*M1AAP and *Pf*M17LAP were located in the cytoplasm, with no overlap with either the DV or the nucleus but possibly associated with (unidentified) punctate structures (Supplementary Figs. 3 and 4).

### Visualization of aminopeptidase activity in live cells using fluorogenic peptide substrates

**Figure 6:**
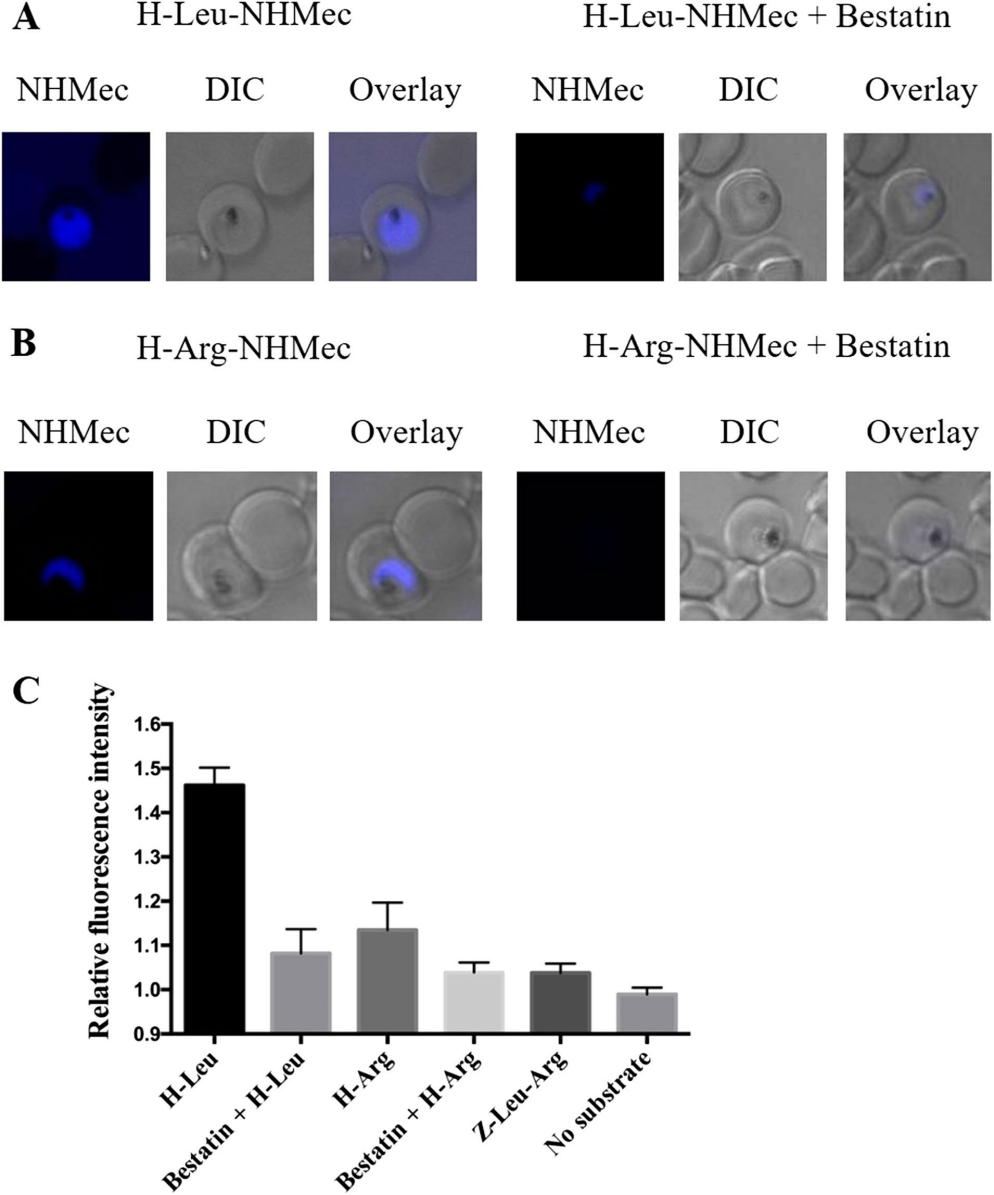
Proteolytic cleavage of fluorogenic substrates by *Pf*M1AAP and *Pf*M17LAP in the cytosol of live parasites. Erythrocytes infected with 3D7 *P. falciparum* parasites were incubated with either (**A**) 10 µM H-Leu-NHMec (substrate cleaved by *Pf*M1AAP and *Pf*M17LAP) or (**B**) 10 µM H-Arg-NHMec (substrate cleaved by *Pf*M1AAP but not *Pf*M17LAP) for 10 min. Proteolytic cleavage of substrates resulted in the release of the fluorescent free NHMec fluorophore in the parasite cytosol (blue staining). Previous incubation with 50 µM bestatin, an aminopeptidase inhibitor, resulted in inhibition of aminopeptidase activity, as revealed by the almost complete absence of fluorescence. Differential interference contrast (DIC) is shown for reference. (**C**) Quantification of NHMec fluorescence intensities in the cytosol relative to background (mean ± SEM). At least ten parasites were imaged per treatment under the same conditions on the same day. Z-Leu-Arg-NHMec (substrate not cleaved by *Pf*M1AAP or *Pf*M17LAP) was used for comparison.

We developed a method to visualize active aminopeptidase activity within live *P. falciparum* 3D7 parasites by probing infected erythrocytes with the substrates H-Leu-NHMec or H-Arg-NHMec which we have shown are specific for neutral aminopeptidases (^27^; Supplementary Fig. 8). For this, parasite-infected erythrocytes were incubated with either 10 µM H-Leu-NHMec or H-Arg-NHMec. The release of the blue-fluorescent fluorophore NHMec at the cellular site where the substrate was cleaved was monitored for 10 min. With both substrates, fluorescence was observed in the parasite cytoplasm, indicating the presence of functional aminopeptidase in this cellular compartment (Fig. 6A,B). Stronger NHMec fluorescence was observed in parasites that were incubated with H-Leu-NHMec compared to those treated with H-Arg-NHMec, which can be explained by the fact that the former substrate is cleaved by both *Pf*M1AAP and *Pf*M17LAP aminopeptidases, whereas the latter is only cleaved by *Pf*M1AAP. Pre-incubation for 5 min with 50 µM bestatin, an inhibitor of both *Pf*M1AAP and *Pf*M17LAP activity, inhibited the release of NHMec from H-Arg-NHMec and H-Leu-NHMec (Fig. 6A,B); analysis of the intensity of fluorescence within the cytoplasm of treated and non-treated parasites (at least ten parasites each) indicated a reduction in cytoplasmic NHMec fluorescence intensity by 66% and 80%, respectively (Fig. 6C). In contrast, treatment with Z-Leu-Arg-NHMec, an N-terminally blocked substrate not cleaved by aminopeptidases showed only slightly higher fluorescence within the cytoplasm compared to background (Fig. 6C; Supplementary Fig. 8).

### pH optimum for *Pf*M1AAP and *Pf*M17LAP activity

The pH of the malaria parasite cytosol is estimated to be neutral at approximately 7.2, while the digestive vacuole operates at an acidic pH of 5.2–5.5^33,34^. In order to assess whether *Pf*M17LAP and *Pf*M1AAP enzymes are functional at these pH conditions, we performed enzyme assays using a range of pH buffers (pH 4–9). 100 mM NaCl was added to each buffer to equalise their ionic strength.

**Figure 7:**
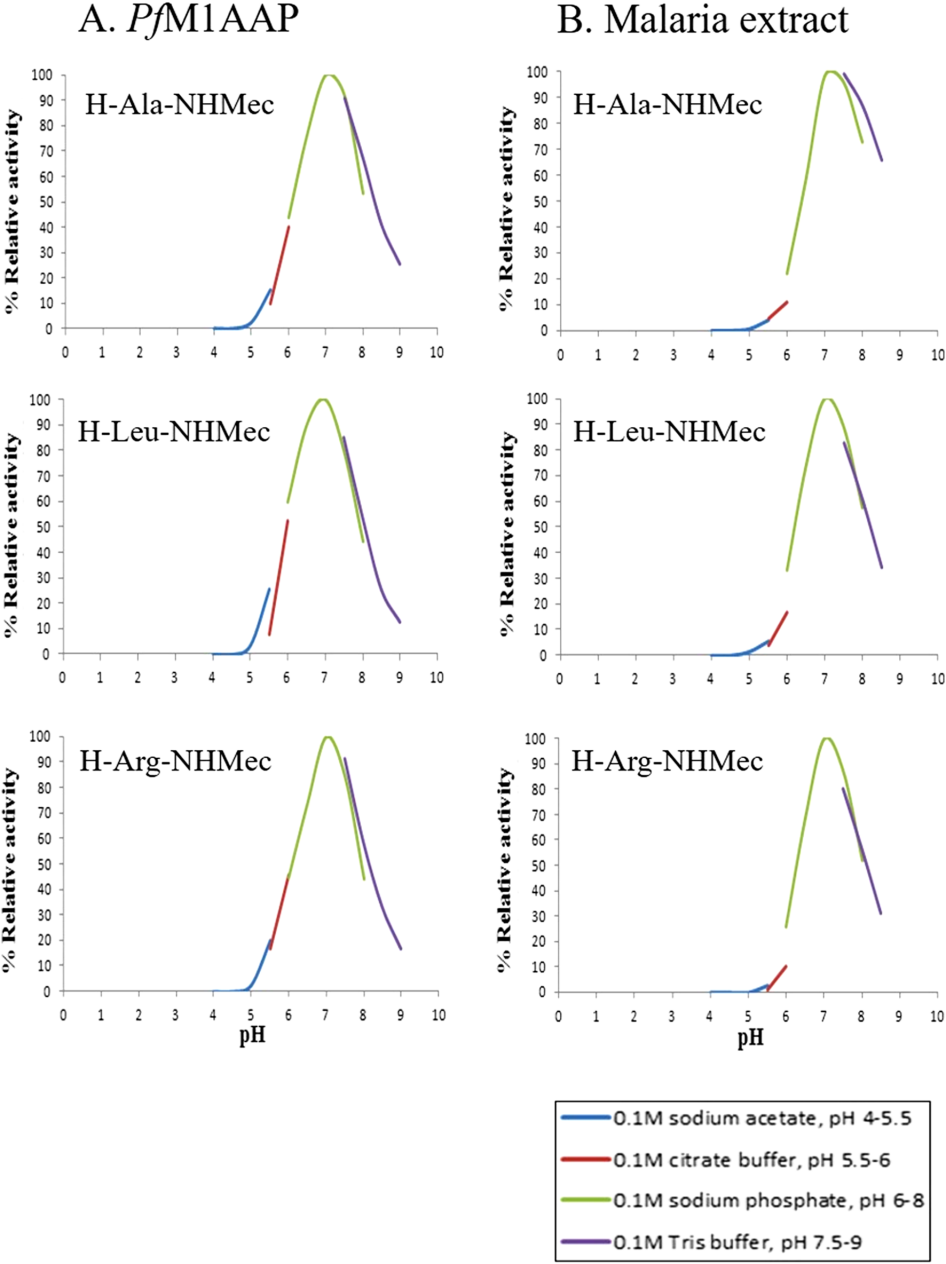
pH profiles of *Pf*M1AAP and soluble extracts of malaria parasites. Recombinant *Pf*M1AAP enzyme (1 μg) (**A**) or soluble extract of malaria parasites (5 μg) (**B**) was incubated in a series of pH-controlled buffers (0.1 M sodium acetate pH 5.0–5.5, 0.1 M sodium citrate pH 5.5–6.0, 0.1 M sodium phosphate pH 6.0–8.0, 0.1 M Tris buffer pH 7.5–8.5) in the presence of the fluorogenic peptide substrates H-Ala-NHMec, H-Leu-NHMec or H-Arg-NHMec. The release of the fluorophore NHMec was monitored in a fluorimeter with excitation at 360 nm and emission at 460 nm. Reaction rates were used to plot pH-activity curves.

As previously reported^6^, *Pf*M17LAP exhibited the classic pH profile of a cytoplasmic enzyme, i.e. optimal pH in the neutral range pH 7.0–8.0 and no activity below pH 6.0 (see Supplementary Fig. 5). By contrast, *Pf*M1AAP showed a much broader pH profile ranging from pH 5.0 to pH 9.0, and peaking at pH 7.0 (Fig. 7A). Similar pH profiles were observed for the aminopeptidase activity measured in the malaria cytosolic extract using the substrate H-Ala-NHMec, that is preferentially cleaved by *Pf*M1AAP compared to *Pf*M17LAP, and with H-Arg-NHMec, which is exclusively cleaved by *Pf*M1AAP^6,7^ (Fig. 7B).

**Figure 8:**
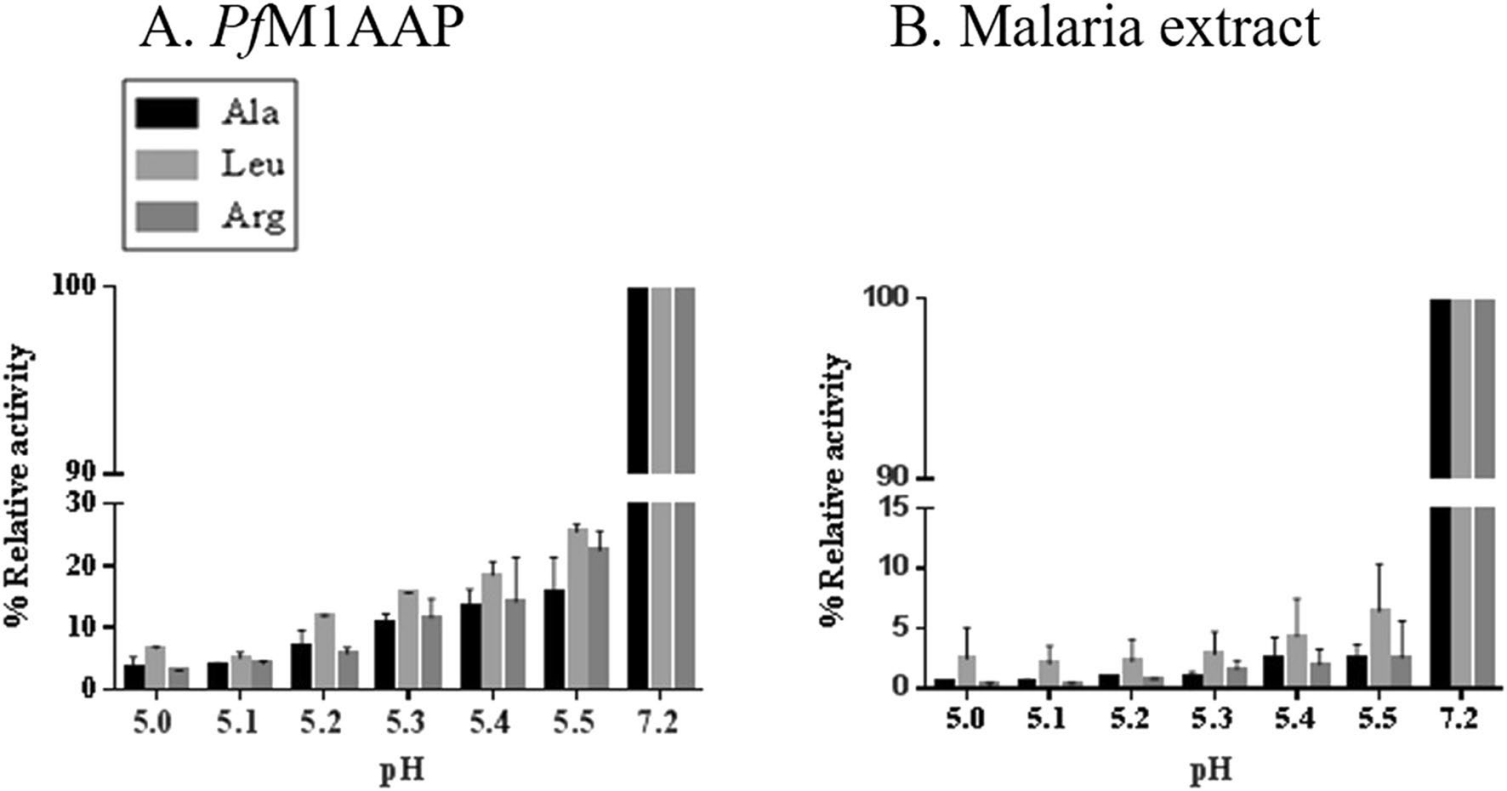
Activity profile *Pf*M1AAP at low pH. Aminopeptidase activity of (**A**) recombinant *Pf*M1AAP and (**B**) soluble extracts of *P. falciparum* malaria parasites in the acidic pH range of 5.0–5.5 (0.1 M sodium acetate buffer) compared with neutral pH 7.2 (PBS). The preparations were incubated in the buffers just prior to the addition of 10 μM substrates (H-Ala-NHMec, H-Leu-NHMec and H-Arg-NHMec).

Further experiments were performed to examine the activity of recombinant *Pf*M1AAP and aminopeptidase activity in parasite cytosolic extracts in the acidic pH range from 5.0 to 5.5, which broadly simulates that found in the parasite DV^33,34^ (Fig. 8A). For comparison, we used physiological buffered saline (PBS) buffer to represent the activity of the enzyme at the neutral pH 7.2. The relative activity of the recombinant *Pf*M1AAP in the acidic pH range was less than 20% of the activity that was observed at pH 7.2 (Fig. 8A). In *P. falciparum* cytosolic extracts, the aminopeptidase activity at acidic pH was less than 5% of that determined at pH 7.2 (Fig. 8B).

The kinetic parameters K_m_, K_cat_, K_cat_/K_m_ were determined for recombinant *Pf*M1AAP against the fluorogenic substrates H-Ala-NHMec, H-Leu-NHMec and H-Arg-NHMec at pH 7.5 and pH 5.2. Consistent with earlier studies, the Michaelis Menten constant (K_m_) for these fluorogenic substrates at neutral pH is relatively high (> 197 µM). However, the K_m_ values obtained for pH 5.2 were between three and nine times higher than that observed at pH 7.5. The catalytic efficiency of recombinant *Pf*M1AAP for each fluorogenic substrate, estimated using the parameter K_cat_/K_m_, were 5.6–15.9 times higher at pH 7.5 compared to pH 5.2 (Table 2).

**Table 2:** Enzyme kinetics studies with *Pf*M1AAP.

Substrate	pH	K_m_ (µM)	K_cat_ (s^−1^)	K_cat_/K_m_ (M^−1^S^−1^)	K_cat_/K_m_ (7:5/5:5 ratio)
Ala	7.5	197	0.9	4394.5	15.9
	5.2	1276	0.4	276.7	
Leu	7.5	197	0.8	4139.4	10.2
	5.2	1714	0.7	407.3	
Arg	7.5	308	0.7	2335.3	5.6
	5.2	980	0.4	420.3	

## Discussion

The widely accepted model for haemoglobin degradation by malaria parasites involves the breakdown of haemoglobin to haem and globin within the DV by proteases of various mechanistic classes. The haem moiety is converted to an insoluble crystalline form, called hemozoin, while globin is degraded by the concerted action of endopeptidases (plasmepsins, falcipains and falcilysin) and exo-peptidases (dipeptidyl aminopeptidases) to generate peptides and amino acids^35–38^. We have suggested that the peptides are then transported from the DV into the cytoplasm, where they are hydrolysed by aminopeptidases, such as *Pf*M1AAP and *Pf*M17LAP, releasing amino acids that are then used by the rapidly growing parasite for protein synthesis^6,9,13^. Both aminopeptidases are maximally expressed in early trophozoites^9^, though Harbut et al.^39^ showed that *Pf*M17LAP is also expressed in the early stages of the intra-erythrocytic parasite and suggested that this aminopeptidase may have an additional role prior to the onset of haemoglobin digestion. Recent studies, however, suggest that haemoglobin digestion already begins at the early ring stage [^40,41^, reviewed in^42^].

In this study, immunoblot analysis of cell fractions using specific anti-*Pf*M1AAP and anti-*Pf*M17LAP antibodies shows that most of *Pf*M1AAP and all of *Pf*M17LAP is found in the cytosolic fractions (C1 and C2). This correlated with the observation that most aminopeptidase activity (> 90%), measured using different fluorogenic peptide substrates of aminopeptidases, was associated with this cytosolic fraction. While *Pf*M17LAP was detected in the cytosolic fraction as a single parent protein of 58 kDa, *Pf*M1AAP was observed as a full-length 115-kDa parent molecule and a 70-kDa fragment. Only a small amount of the 70-kDa fragment was associated with a vacuolar fraction, V1, and may be loosely bound to the membrane as a peripheral protein, since it was extracted from this fraction by freeze–thaw. Plasmepsin I, a soluble DV aspartic protease, is also associated with this fraction.

Azimzadeh et al.^28^ reported that *Pf*M1AAP appeared in soluble extracts of *P. falciparum* as three forms with molecular weights of 68 kDa, 96 kDa and 120 kDa. Densitometric measurements performed by this group calculated that 16% of the 68-kDa form is associated with a DV fraction^28^. Follow-up studies by Allary et al.^11^ showed that the 120-kDa form of *Pf*M1AAP appeared only in extracts prepared in the presence of protease inhibitors, while a 115-kDa protein species was detected in the absence of protease inhibitor. However, the 68-kDa form was present in both preparations of the cell extracts. In our studies, we observed a ~ 70-kDa and ~ 115-kDa band in the cytosolic fraction prepared in the presence of protease inhibitors and a ~ 70-kDa and ~ 100-kDa band in the cytosolic fractions prepared in the absence of protease inhibitors. Our results mostly agree with the observations of Azimzadeh et al.^28^ and Allary et al.^11^; the differences we describe in molecular sizes of the various molecular forms of *Pf*M1AAP likely result from a difference in the gel and molecular size marker systems employed. It is important to note that, in our study, we purposely employed molecular size markers and a gel system to optimize protein separation in the 60–120 kDa range. Hence, we speculate that the 68-kDa, 115-kDa and 120 kDa fragments described earlier by these authors represent the 70-kDa, 100-kDa and 115-kDa fragments observed in this study.

We have established that the 115-kDa parent form of *Pf*M1AAP is proteolytically cleaved to the 100-kDa form in soluble extracts prepared in the absence of protease inhibitors. The nature of the protease responsible for this hydrolysis is not known. The soluble parasite extract could contain several proteases, including cysteine proteases (falcipains) and metalloproteases (falcilysin), as well as other potential serine proteases involved in protein processing and cell rupture^35,36,43^. However, when cytosolic fractions prepared with and without protease inhibitors were incubated overnight at 37 °C, the ~ 70-kDa and ~ 100-kDa forms of *Pf*M1AAP appeared in both preparations. Only an additional band of ~ 65 kDa was found in the cytosolic fractions prepared without a protease inhibitor. The data suggests that the formation of the ~ 70-kDa and ~ 100-kDa forms result from specific, rather than random, proteolytic cleavages. Immunoblots probed with antibodies prepared to the N-terminal extension (anti-PepA), middle domain (anti-PepB) and C-terminal domain (anti-PepC) show that the hydrolytic event that gives rise to ~ 100-kDa form involves removal of the N-terminal extension. By contrast, the 70-kDa form lacks the N-terminal extension and a C-terminal extension in keeping with the observations of Allary et al.^11^. The addition of soluble extracts of malaria parasites to our recombinant *Pf*M1AAP did not produce a 70-kDa fragment, which suggests that the proteolytic enzyme responsible for this processing event is located at a specific site within the cell, is not extracted in sufficient quantities into the cytosolic extract and/or is instable.

Primary sequence analysis of *Pf*M1AAP shows that the 70-kDa, 100-kDa and 115-kDa forms all contain the active site residues within the catalytic domain (domain 2) comprising of the canonical H^496^EYFHX_17_KE^519^ signature of the M1 family zinc-metallopeptidases, as well as the conserved GAMEN motif that is involved in substrate specificity. However, the ~ 70-kDa fragment lacks the C-terminal domain (domain 4), which 3-D structure analysis shows is critical in creating a 30-Å long channel that permits substrates access to the buried active site^7^. A shallow 8-Å long groove is also formed at the junction of domains 1 and 4 and allows the exit of released amino acids^7^. Moore et al.^44^ observed that the migration of the substrates and inhibitors along the 30-Å long channel involves several transient states which are stabilized by interactions with specific residues of the domain, including the positively charged residues Arg^969^, Arg^489^, Lys^849^, Lys^907^, and negatively charged residues Glu^850^, Asp^830^, Glu^572^ and Asp^581^. Furthermore, in the 3D structure of *Pf*M1AAP complexed with the inhibitors bestatin and compound 4, the residue Met^1034^ in domain 4 was observed to be involved in making optimal contacts with the ligands^7^. The consequence of this analysis is that, since the ~ 70-kDa fragment of *Pf*M1AAP is missing many essential structures and residues for aminopeptidase function, it is highly unlikely that it is enzymatically active and, hence, its role in the parasite is an enigma. A recombinant version of the ~ 70-kDa fragment prepared in our laboratory did not exhibit any aminopeptidase activity (Supplementary Fig. 6).

The intra-cellular locations of *Pf*M1AAP and *Pf*M17LAP were examined by immuno-fluorescence analysis using several fixation conditions aimed at preserving the integrity of the different cellular compartments. Irrespective of the fixation conditions employed, both *Pf*M1AAP and *Pf*M17LAP were restricted to the cytoplasm with no strong evidence of substantial localisation of either enzyme within the DV or nucleus (as expected, the control anti-plasmepsin I antibody localised to the parasite DV^26^). With some fixation procedures, the enzyme localisation appeared punctate, the relevance of which is not fully understood at present (Supplementary Figs. 3 and 4). We also showed that fluorogenic peptides that are substrates for *Pf*M1AAP (H-Arg-NHMec and H-Leu-NHMec) and *Pf*M17LAP (H-Leu-NHMec) can passively enter live *P. falciparum*-infected erythrocytes and allow visualisation of functional aminopeptidase inside the parasite; the results demonstrated aminopeptidase activity within the cytoplasm, and this was inhibited by the aminopeptidase-specific inhibitor bestatin that we previously reported can kill malaria parasites in culture^6,7^.

The sub-cellular compartment where *Pf*M1AAP is functionally active in the degradation of haemoglobin peptides has been a subject of debate due to conflicting data regarding its localisation. Using live parasites transfected with GFP-tagged constructs, we previously reported that *Pf*M1AAP was located in the parasite cytosol^7^. Studies by Allary et al.^11^ also suggested that all three forms of *Pf*M1AAP (68 kDa, 96 kDa and 120 kDa) localised within the cytosol; no localisation was observed within the DV, although distinct accumulation around the DV was described. The same laboratory described how the 96-kDa *Pf*M1AAP is trafficked outside the parasite into the parasitophorous vacuolar (PV) space before being converted to the 68-kDa form and re-directed into the parasite cytoplasm and partly (16%) to the DV^28^. In contrast, studies by Dalal and Klemba^14^ and Ragheb et al.^29^ using *P. falciparum* parasites that stably expressed YFP-tagged *Pf*M1AAP, localised this enzyme to the DV (more prominently, at the rim of the organelle) and nucleus but not in the cytoplasm. These results led Klemba et al. to propose that *Pf*M1AAP operates within the DV, downstream of endopeptidases (falcipain, plasmepsins and dipeptidase), to release free amino acids, and has a separate, as yet unknown, function within the nucleus. Biochemical data showing that *Pf*M1AAP was active and stable at pH 5.5^29^, and observations of DV swelling in parasites incubated with inhibitors of *Pf*M1AAP^39^, added support to the proposed function for this enzyme in the DV.

Since the pH of the DV and cytosol differ significantly, pH, 5.2 and 7.2, respectively^33,34^, we performed experiments to determine the pH optimum for activity of recombinant *Pf*M1AAP and *Pf*M17LAP. *Pf*M17LAP showed a sharp peak at pH 7.0–8.0 and no activity below pH 6.0, which is consistent with its cytoplasmic location and function. By contrast, *Pf*M1AAP exhibited a distinctly different profile, with its activity spreading over a broader pH range, pH 5.0–9.0, although peaking between pH 7.0 and 7.5. Detailed pH-activity kinetic studies using three fluorogenic peptide substrates (H-Ala-NHMec, H-Leu-NHMec and H-Arg-NHMec) determined that the rate of activity of the *Pf*M1AAP was highest at pH 7.0 for all the three peptide substrates, with approximately 20% activity at pH 5.5, and no activity below pH 5.0. Whole cell soluble extracts of malaria parasites exhibited a similar profile that was consistent with our earlier published studies^6,7^.

We performed a closer evaluation of the pH-activity relationship in the acidic pH range 5.0–5.5, which incorporates the reported pH present within the DV^33,34,43^. Both the recombinant *Pf*M1AAP and total aminopeptidases in soluble parasite extracts displayed low activity in the acidic pH range compared to pH 7.2. Therefore, in support of the data of Klemba et al.^29^, the *Pf*M1AAP can function at a pH value between 5.0 and 5.5, even though this is much lower (~ 20%) than the activity at pH 7.2.

The observation that *Pf*M1AAP exhibits a single peak for activity at neutral pH using substrates with hydrophobic (Ala, Leu) and positively charged (Arg) amino acids in the P1 position is relevant because enzymes that function in two different pH environments can often possess different pH optima against different substrates. Such pH switching was reported for the *P. vivax* cysteine protease, vivapain-4 (VX-4), where the pH-activity profile changes from an acidic to a neutral pH optimum when the amino acid of the substrate is changed from a hydrophobic residue (Phe, Leu) to a positively charged residue (Arg)^45^. This behaviour is indicative of the role this peptidase plays as a digestive enzyme in the acidic DV and as a cytoskeleton-degrading peptidase in the pH neutral erythrocyte cytosol^45,46^. Another example is the *P. falciparum* M16 metalloprotease falcilysin, which is located within the DV and other vesicular structures of the parasite^47^ and whose proteolytic activity at neutral pH and acidic pH is substantially dependent on the peptide substrate^47^. Thus, the lack of pH switching observed for recombinant *Pf*M1AAP (and *P. falciparum* cytosolic extracts), indirectly supports the idea that the prime function of the enzyme is at neutral pH.

In summary, our studies strongly support the idea that the *Pf*M1AAP and *Pf*M17LAP enzymes function within the parasite cytosol of *P. falciparum*. *Pf*M1AAP has three different forms with molecular weights of ~ 70 kDa, ~ 100 kDa and ~ 115 kDa. Although a small proportion of the ~ 70-kDa form was visualized in the vacuolar fraction and may associate with the DV, due to its lack of domain 4 we have judged this fragment to be enzymatically inactive. Its processing activity within the malaria cell requires further investigation. Our collective immunolocalization studies show these enzymes to be strictly localized in the cytosol, where they may function in the final stages of haemoglobin digestion. Cytosolic M1AAP and M17LAP, with neutral pH optima, have been characterised in other apicomplexan parasites such as *Babesia* sp.^48,49^, *Eimeria tenella*^50^, and *Toxoplasma gondii*^51,52^; so they clearly perform fundamental processes within these unicellular pathogens making them broadly prominent as anti-parasitic drug targets.

## Methods

### Chemicals and antibodies

All chemicals were purchased from either Sigma-Aldrich (Canada) or Bioshop Inc. (Canada). The fluorogenic peptide substrates H-alanine 7-amido-4-methylcoumarin (H-Ala-NHMec), leucine 7-amido-4-methylcoumarin (H-Leu-NHMec) and H-arginine 7-amido-4-methylcoumarin (H-Arg-NHMec) were obtained from Biosynth AG (Switzerland). The inhibitor bestatin was purchased from Cayman Chemical Company (USA).

### In vitro culture of *Plasmodium falciparum parasites*

*P. falciparum* strain 3D7 was cultured in human A + erythrocytes according to modified standard procedures^53^ at 5% CO_2_ and 3% O_2_ using RPMI 1640 medium supplemented with 0.5% (w/v) Albumax II, 20 mg/ml gentamicin (Life Technologies, Canada) and 100 µM hypoxanthine. Standard static cultures were generally employed except for cellular fractionation experiments, where larger scale cultures (1 l) were prepared in a Wave Bioreactor^54^. For stage-specific *Pf*M1AAP and *Pf*M17LAP expression analysis, the parasites were synchronised using sorbitol treatment^55^.

### Cloning, expression and purification of recombinant *Pf*M1AAP and *Pf*M17LAP

The cDNA encoding the *P. falciparum* M1 alanine aminopeptidase, *Pf*M1AAP, was constructed as described by McGowan et al*.*^7^. The cDNA encoding the *P. falciparum* M17 leucine aminopeptidase, *Pf*M17LAP was constructed as described previously by Stack et al.^6^ and McGowan et al.^13^. Both cDNAs were synthesised by GeneArt GmbH (Germany). The cDNAs were cloned into the expression vector pTrcHis2B (Invitrogen) and *E. coli* strain BL21 (Novagen) was employed as the expression host.

Bacteria were grown at 37 °C in Luria Bertani (LB) medium, supplemented with 100 μg ml^−1^ ampicillin, to an optical density of 0.6 at 600 nm. Recombinant protein production was induced by the addition of 1 mM IPTG for 3 h at 30 °C. The culture broth was centrifuged at 5000 × *g* for 10 min. The pellet was re-suspended in lysis buffer (50 mM NaH_2_PO_4_, 300 mM NaCl, 5 mM imidazole, pH 8.0) and incubated with 1 mg ml^−1^ lysozyme for 30 min on ice. The suspension was sonicated using a sonic dismembrator system (Fisher Scientific, USA) for three cycles of 10 s each with 10 s rest. Crude protein lysate was obtained after centrifugation at 24,000 × *g* for 30 min at 4 °C. The supernatant was passed through a 0.45-μm filter (Corning Inc, USA) to remove cell debris and then diluted five times with lysis buffer.

Both recombinant enzymes were purified by affinity chromatography using Ni–NTA agarose columns (usually 1 ml matrix volume; Qiagen). Columns were washed twice with 10 volumes of wash buffer (50 mM NaH_2_PO_4_, 300 mM NaCl, 10 mM imidazole, pH 8.0) before eluting the enzyme using elution buffer (50 mM NaH_2_PO_4_, 300 mM NaCl, 250 mM imidazole, pH 7.0). In the case of *Pf*M1AAP, the recombinant enzyme was dialyzed against 0.1 M HEPES buffer containing 10 μM ZnCl_2._ For *Pf*M17LAP, the recombinant enzyme was dialysed against 0.1 M HEPES buffer supplemented with 10 μM CoCl_2_. The quality of purification of both the enzymes was analysed using SDS-PAGE electrophoresis and immunoblotting.

### Antibody production and adsorption

Antibodies against *Pf*M1AAP and *Pf*M17LAP were raised in a rabbit using a purified recombinant enzyme as the immunogen (Institute of Medical and Veterinary Science, IMVS, Adelaide, Australia). Monoclonal antibody prepared against the digestive vacuolar plasmepsin I (*Pf*PM1) was obtained from the American type culture collection (MRA-813A, MR4/American Type Culture Collection (ATCC), Manassas, VA, USA).

To prevent non-specific binding on immunoblots, polyclonal antibodies against *Pf*M1AAP and *Pf*M17LAP were adsorbed against extracts of *E. coli* expressing the His-tagged protein SmPrx 1 (peroxiredoxin derived from *Schistosoma mansoni*). The lysate from *E. coli* expressing SmPrx1 was prepared as detailed above. Five nitrocellulose membranes (0.2-µm pore-size, Bio Rad) were cut to size, added to a petri dish containing the 10 ml of the diluted crude protein lysate and incubated with shaking for 1 h at room temperature. The membranes were washed three times for 15 min with Tris-buffered saline/0.05% Tween (TBST) at room temperature to wash off any unbound protein. The anti-*Pf*M1AAP or anti-*Pf*M17LAP serum was diluted in TBST (diluted 1:10,000 and 1:30,000, respectively) and added to separate petri dishes. One washed membrane with bound crude lysate was added at a time to the petri dishes containing the diluted anti-serum and incubated with shaking for 1 h at room temperature. The membrane was discarded at the end of 1 h and the process repeated. After this step, the anti-*Pf*M1AAP and anti-*Pf*M17LAP serum was removed, checked for specificity (Supplementary Fig. 7) and stored in aliquots at − 20 °C until used in immunoblotting or immunocytochemical studies.

Affinity-purified antibodies were prepared against three different 14-mer peptides derived from *Pf*M1AAP (GenScript, USA). The positions of these peptides within the aminopeptidase primary sequence are shown in Supplementary Fig. 1 and are as follows: 140–153 (anti-PepA) within the N-terminal extension, 366–380 (anti-PepB) within Domain 1 and 873–889 (anti-PepC) within Domain 4.

### Fractionation of *P. falciparum* cell compartments

Cellular fractions of *P. falciparum* were prepared using a modified method of Saliba et al.^25^ and carried out as the flowchart in Fig. 1. About 2 ml packed cell volume (PCV) of erythrocytes at 5–7% parasitaemia were washed three times with ice-cold phosphate buffered saline (PBS) in a 50-ml Falcon tube. Parasites were isolated from erythrocytes by resuspending the cells in 7 ml 0.05% saponin/PBS and incubating on ice for 5 min. The freed parasites were washed three times with ice-cold PBS and finally resuspended in ten volumes of ice-cold 10 mM Tris–HCl buffer, pH 7.2. A sample of the suspension was removed to prepare total soluble parasite extract (TPE) while the remaining parasite cells were triturated four times through a 27G × 1/2 needle (BD, USA) and then centrifuged at 17,500 × *g* for 10 min at 4 °C. The supernatant, termed the first cytosolic fraction (C1), was removed and stored at − 20 °C. The pellet was suspended to the same volume in ice-cold 10 mM Tris–HCl buffer and the process repeated, resulting in a supernatant termed the second cytosolic fraction (C2), which was removed and stored at − 20 °C. The pellet was re-suspended again to the same volume in ice-cold 10 mM Tris–HCl and subjected to four rounds of freeze–thaw and sonication. This suspension was centrifuged at 17,500 × *g* for 10 min at 4 °C. The supernatant, termed the soluble vacuolar fraction (V1), was removed and stored at − 20 °C. The pellet was re-suspended in 0.5% Triton X in 10 mM Tris–HCl and kept on ice for 30 min before being centrifuged at 17,500 × *g* for 10 min at 4 °C to obtain the second vacuolar fraction (V2).

To study the effect of protease inhibitor on the processing of *Pf*M1AAP, saponin-released parasites were divided into equal parts. One part was suspended in ten volumes of ice-cold 10 mM Tris–HCl buffer, pH 7.2, and the other suspended in the same buffer supplemented with protease inhibitor cocktail tablets (Roche Diagnostics, Canada). The cytosolic fraction (C1) was prepared for both parasite suspensions by trituration four times through a 27G × 1/2 needle as described above. Some samples were frozen immediately at − 20 °C while others were incubated at 37 °C in a water bath overnight. Immunoblots were performed using anti-*Pf*M1AAP and anti-*Pf*M17LAP prepared as described below.

To determine if the C1 extract of *P. falciparum* parasites contained enzymes capable of hydrolysing recombinant *Pf*M1AAP, this protein (0.5 µg) was incubated with cytosolic fractions (5 µg), extracted with and without protease inhibitors, at 37 °C overnight. Samples were then subjected to immunoblotting analysis using anti-His-tag mouse antibody primary antibody (1:1500 dilution) and peroxidase-conjugated anti-mouse secondary antibody (1:1500 dilution).

### Immunoblotting

Parasite fractions separated by SDS-PAGE electrophoresis were transferred to nitrocellulose membranes using a semi-dry transfer cell (Trans-blot, Bio Rad). The membranes were blocked with 5% skim milk in Tris-buffered saline containing 0.1% Tween 20 (TBST) for 2 h and then probed with anti-*Pf*M1AAP and anti- *Pf*M17LAP antibodies in 2.5% skim milk (diluted 1:10,000 and 1:30,000, respectively). Samples were also probed with anti-plasmepsin I monoclonal mouse antibody at a dilution of 1:5000 in 2.5% milk in TBST. The membranes were probed at 4 °C overnight, washed three times in 20 ml TBST for 15 min at room temperature and then incubated in horseradish peroxidase-conjugated anti-rabbit IgG diluted in 2.5% milk in TBST (1:25,000 dilution for anti-*Pf*M1AAP antibodies and 1:50,000 dilution for anti-*Pf*M17LAP antibodies) (ThermoFisher Scientific, USA). A 1:1500 dilution of anti-mouse secondary antibody was used for detecting plasmepsin I. Bound antibody was visualized by the addition of 1 ml of SuperSignal West Femto Chemiluminescent substrate (ThermoFisher Scientific, USA) for 5 min. Chemiluminescent signal was developed using the autoradiography cassette and Kodak X-OMAT 2000 processor system. Chemiluminescent molecular size markers (Invitrogen, Canada) were employed to visualise standards directly on film.

### Immunofluorescence assays

Immunofluorescence assays were carried out as described previously using air-dried *P. falciparum-*infected red blood cells fixed with 4% paraformaldehyde (PFA) and 0.0075% glutaraldehyde (Electron Microscopy Sciences, USA) for 20 min at room temperature and permeabilized with 0.5% Triton X-100 (Bioshop Canada Inc., Canada) for 10 min^14,28,29^. Alternatively, parasites were fixed and permeabilized with a mixture of 75% acetone and 25% methanol (VWR International Co., Quebec, Canada) for 5 min at − 20 °C^10,11,28^ or of 50% ethanol and 50% methanol for 2 min at − 20 °C^11^. The parasites were blocked for 2 h using 3% bovine serum albumin (BSA, Sigma-Aldrich) in PBS and then probed overnight at 4 °C with polyclonal antibodies against *Pf*M1AAP (1:500) or *Pf*M17LAP (1:500) raised in rabbits. These parasites were then subsequently probed with mouse polyclonal antibodies against *Pf*M1AAP (1:20 dilution) for 4 h at 4 °C, washed with PBS and then incubated for 1 h with Alexa-Fluor 488 goat anti-rabbit and Alexa-Fluor 594 goat anti-mouse (1:1000, Life Technologies). The parasite nuclei were visualized with 500 nM DAPI (Life Technologies). After removal of unbound antibodies and DAPI by washing with PBS, the slides were mounted with coverslips using ProLong Gold antifade reagent (Life Technologies) and kept at 4 °C until evaluation. Samples were viewed on an LSM710 confocal microscope system (Carl Zeiss GmbH, Germany) with a 100×/1.4 oil objective. The 405-nm, 488-nm and 594-nm laser lines were used for excitation and the captured images were processed using ImageJ 1.53c (National Institutes of Health, http://rsb.info.nih.gov/ij) with the plugin “coloc 2” for colocalization assessment.

### Live-cell imaging

Erythrocytes infected with *P. falciparum* 3D7 parasites were washed twice with Ringer’s solution (122.5 mM NaCl, 5.4 mM KCl, 1.2 mM CaCl_2_, 0.8 mM MgCl_2_, 11 mM D-glucose, 25 mM HEPES, 1 mM NaH_2_PO_4_, pH 7.4) and incubated with either 10 µM H-Leu-NHMec or 10 µM H-Arg-NHMec for 10 min in the presence or absence of 50 µM bestatin. As a control, parasites were also incubated with the N-terminally-blocked N-carbobenzyloxy (Z)-Leu-Arg-NHMec that is not cleaved by either aminopeptidase. Parasites were imaged on glass slides using an Imager M2 Axio (Carl Zeiss GmbH) with a Plan-Apochromat 100×/1.4 oil objective, an ET-DAPI 49000 filter set and a Hamamatsu C10600-10B ORCA-R^2^ camera or in an open chamber using a NIKON Eclipse TE2000-U inverted epifluorescence microscope with a Plan-Apochromat 100×/1.4 oil objective and a Hamamatsu 1394 ORCA-ERA camera.

Images of at least ten individual parasites were taken per treatment using the same microscope settings and the same solutions under physiological conditions. The relative fluorescence intensities within the parasite cytosol were compared with an area void of cells in the DIC channel using ImageJ 1.53c. The fluorescence intensities within the respective regions of interest in the DAPI channel were then used to calculate mean background-corrected fluorescence intensities.

### Enzymatic assays

Enzymatic assays were used to quantify the distribution of aminopeptidase activity in different parasite cell fractions. Since the amino acid arginine is uniquely cleaved by *Pf*M1AAP, the fluorogenic peptide substrate H-Arg-NHMec was used for its measurement^6,7^. As no specific substrate is available for *Pf*M17LAP, H-Leu-NHMec, which detects both aminopeptidases, was used. Substrate concentration (10 µM) was well below the K_m_ of each enzyme to ensure that relative activity is proportional to catalytic efficiency of the enzyme (K_cat_/K_m_)^56^. All reactions were initiated by the addition of enzyme to the substrate solution in a reaction volume of 200 µl in 96-well microtitre plates (Corning Inc, USA) at 37 °C. The release of the fluorophore (NHMec) was measured using a spectrofluorometer (Bio-Tek Synergy H1) with an excitation set at 370 nm and emission at 460 nm. Initial rates were obtained from the linear portion of the progress curves using the Gen5 software. A standard graph was plotted using increasing concentrations of free NHMec (0.1–10 µM) and used to determine units of enzyme in each fraction. One unit of enzyme activity is defined as the amount (nanomoles) of NHMec released per 30 min.

To determine pH optima for activity for both recombinant *Pf*M1AAP and *Pf*M17LAP and the native aminopeptidases in the cytosolic malaria parasite extract, these were incubated in buffers (100 mM) of varying pH (sodium acetate buffer, pH 5.0–5.5; sodium citrate buffer, pH 5.5–6.0; sodium phosphate buffer, pH 6.0–8.0; Tris buffer, pH 7.5–8.5) containing 150 mM NaCl to normalise their ionic strength. Each sample was assessed using three fluorogenic substrates, namely H-Ala-NHMec, H-Leu-NHMec and H-Arg-NHMec.

For experiments designed to examine the activity of *Pf*M1AAP at the pH expected for the parasite digestive vacuole^33,34^ assays were performed in pH range 5.0–5.5 using 100 mM sodium acetate buffer containing 150 mM NaCl. In these experiments, PBS with a pH of 7.2 was employed to monitor the activity of the enzyme in the neutral pH range. The assay was performed using three substrates H-Ala-NHMec, H-Leu-NHMec and H-Arg-NHMec at a concentration of 10 µM. In addition, the kinetic parameters of the recombinant enzyme against the three fluorogenic substrates were determined at pH 5.2 and pH 7.5. Initial rates were obtained over a range of substrate concentrations spanning the K_m_ and fitted to the Michaelis–Menten equation. The kinetic parameters were obtained using the SigmaPlot software.

## Supplementary Information

Supplementary Figures.

## Abbreviations

DV: Digestive vacuole
PV: Parasitophorous vacuole
NHMec: 7-Amino-4-methylcoumarin
