# Supplementary material for: Biochemical and cellular characterisation of the Plasmodium falciparum M1 alanyl aminopeptidase (PfM1AAP) and M17 leucyl aminopeptidase (PfM17LAP)
Source: Sci Rep. 2021 Feb 3;11:2854. doi: 10.1038/s41598-021-82499-4 (PMC7858622; doi:10.1038/s41598-021-82499-4)

Biochemical and cellular characterisation of the *Plasmodium falciparum* M1 alanyl aminopeptidase (*Pf*M1AAP) and M17 leucyl aminopeptidase (*Pf*M17LAP).

Rency Mathew, Juliane Wunderlich, Karine Thivierge, Krystyna Cwiklinski, Claire Dumont, Leann Tilley, Petra Rohrbach, John P. Dalton

Supplementary Information

Supplementary Figure 1. Preparation of antibodies to N-terminal, middle Domain 1 and C-terminal domain of *Pf*M1AAP. (A) Antibodies were prepared against 14-mer peptide sequences at positions 140 – 153 (anti-PepA, yellow) within the N-terminal extention, 366-380 (anti- PepB, magenta) within Domain 1 and 873 – 889 (anti-PepC, green) within C-terminal Domain 4. Glutaminic acid (E) 195 is the first residue in the recombinant form of *Pf*M1AAP. The start and end of the p68 processed form (~70 kDa) of *Pf*M1AAP according to the paper by Azimzadeh *et al*. [27] are indicated by the boxed and in bold **VKKNE** and **PVNAQ** sequences, respectively. **(B)** Schematic representation of the sequence and position of PepA, PepB and PepC within the *Pf*M1AAP.

Supplementary Figure 2. Incubation of recombinant *Pf*M1AAP with *P. falciparum* cytosolic fraction (C1). Recombinant *Pf*M1AAP (1 μg) was incubated overnight (16 h) with cytosolic C1 fractions (5 μg) that were prepared without (-) or with (+) a cocktail of proteinase inhibitors. Samples taken before (lanes 2 and 3) and after (lanes 4 and 5) the overnight incubation were analysed by immunoblots probed with anti-His-tag mouse monoclonal antibodies. The 100-kDa recombinant *Pf*M1AAP enzyme is resistant to hydrolysis by the cytosolic C1 fractions.

Supplementary Figure 3. IFA using paraformaldehyde/glutaraldehyde fixed cells malaria-infected erythrocytes. Concanavalin A (0.5 mg/ml) was coated onto a coverslip in humid chamber at 37°C for 30 minutes. iRBCs were harvested, washed in PBS and resuspended in PBS at 3% hematocrit and incubated for 15 minutes at RT on the coverslip. Cells were fixed with 2% PFA and 0.008% glutaraldehyde for 20 minutes at RT. Cells were permeabilised by adding PBS 0.1% Triton X-100 for 10 minutes at RT. Then, 3% BSA in PBS was added and incubated for 1 hour at RT as blocking step before adding the primary antibody diluted in PBS/3% BSA and

incubate for 1 hour at RT. The secondary antibody was diluted in PBS/3% BSA, and incubated for 1 hour at RT. DAPI (10 µg/ml in PBS) was added. Parasite nuclei were visualized using DAPI (blue staining; 4,6-diamidino-2-phenylindole). Red staining highlights the **(A)** cells probed with anti-*Pf*M1AAP antibodies and **(B)** cells probed with anti-*Pf*M17LAP antibodies. Panel A, differential interference contrast (DIC) is shown for reference; Panel B, cells probed with antibodies; Panel C, merged.

Supplementary Figure 4. IFA using acetone-fixed malaria-infected erythrocytes. iRBCs were harvested, washed in PBS and resuspend in PBS at 3% haematocrit. The iRBC were smeared on a coverslip, air-dried at RT and fixed in acetone (100%) for 10 minutes at RT. PBS/3% BSA was added and incubated for 1 hour at RT as blocking step before adding the primary antibody diluted in PBS/3% BSA and incubating for 1 hour at RT. The secondary antibody was diluted in PBS/ 3% BSA, and incubated for 1 hour at RT. The nucleus was stained with DAPI (10 µg/ml) diluted in PBS. Parasite nuclei were visualized using DAPI (blue staining; 4,6-diamidino-2-phenylindole). Red staining highlights the **(A)** cells probed with anti-*Pf*M1AAP antibodies and **(B)** cells probed with anti-*Pf*M17LAP antibodies. Panel A, differential interference contrast (DIC) is shown for reference; Panel B, cells probed with antibodies; Panel C, merged.

Supplementary Figure 5. pH profile of recombinant *Pf*M17LAP. The pH profile of recombinant *Pf*M17LAP (1 µg) was obtained by incubating the enzyme in a series of pH-controlled buffers that were also employed for the studies of the *Pf*M1AAP in this report; these were 0.1M sodium acetate pH 4.0 – 5.5, 0.1M sodium citrate pH 5.5 – 6.0, 0.1 M sodium phosphate pH 6.0 – 8.0, 0.1 M Tris buffer pH 7.5 – 9.0. Reactions were initiated by the the addition of the fluorogenic substrate H-Leu-NHMec at 37°C and the release of the fluorophore NHMec monitored in a fluorimetre set with excitation at 360 nm and emission at 460 nm.

Supplementary Figure 6. Recombinant production of ~70 kDa fragment of *Pf*M1AAP. (A) *E. coli*-expressed recombinant ~70 kDa form of *Pf*M1AAP was partially purified by Ni-Chelate affinity chromatography. The partially purified protein migrated at 70 kDa when analyzed on 10% SDS-PAGE gels. Lane 1: molecular weight standards. Lane 2: insoluble *E. coli* lysate fraction. Lane 3: soluble *E. coli* lysate fraction. Lane 4: column run-through. Lane 5: column

wash. Lane 6: eluted recombinant *Pf*M1AAP. **(B)** Immunodetection of recombinant ~70-kDa form of the enzyme using anti-His tag mouse monoclonal antibody (lane 3) and anti-Pep B antibody (lane 4). Molecular weight standards are shown in lane 1.

Supplementary Figure 7. Specificity of antibody against *Pf*M1AAP and *Pf*M17LAP. Antibodies prepared in rabbits against *Pf*M1AAP and *Pf*M17LAP were adsorbed against freeze-thaw/sonicated extracts of *E. coli* expressing a recombinant His-tagged *Schistosoma mansoni* peroxiredoxin (SmPrx) as described in Materials and Methods. Immunoblots were prepared with molecular size markers (lane 1), recombinant *Pf*M1AAP (lane 2) and recombinant *Pf*M17LAP (lane 3) and probed with the adsorbed anti-*Pf*M1AAP (Panel A) or anti-*Pf*M17LAP (Panel B). These adsorbed antibodies were employed in both immunoblot and cytochemical studies in this report.

Supplementary Figure 8. Relative activity of *Pf*M1AAP, *Pf*M17LAP and *P. falciparum* 3D7 parasite cytosolic extract towards several fluorogenic NHMec substrates (20 μM) at pH 7.3. Enzyme assays were performed as described in Materials and Methods. Results shown are the average of three replicates in a typical substrate screening. The substrates used were Leu, H-Leu-NHMec; Glu, H-Glu-NHMec; Arg, H-Arg-NHMec; Pro, H-Pro-NHMec; PR, Pro-Arg-NHMec; ZPR, Z-Pro-Arg-NHMec; TGPR; Tosyl-Gly-Pro-Arg-NHMec; TGPK, Tosyl-Gly-Pro-Lys-NHMec; FR, Phe-Arg-NHMec; ZFR, Z-Phe-Arg-NHMec; ZLR, Z-Leu-Arg-NHMec; ZR, Z-Arg-NHMec.

Supplementary Figure 9. Full length gel photos. (A) Fig. 1B. Immunoblot of the recombinant *Pf*M1AAP and *Pf*M17LAP proteins and *P. falciparum* TPE and cellular fractions (C1, C2, V1 and V2) probed with (i) anti-*Pf*M1AAP, (ii) anti-*Pf*M17LAP, and (iii) anti-plasmepsin 1 antibodies. **(B)** Fig. 3A. Recombinant *Pf*M1AAP was probed with antibodies prepared against 14-mer peptides derived from the N-terminal extension (PepA), spanning domain 1 (PepB) and domain 4 (PepC) and Anti-*Pf*M1AAP. The light grey box denotes lanes that contain proteins not relevant to this study. **(C)** Fig. 3B. Cytosolic fractions of malaria parasites prepared without (-) and with (+) a protease inhibitor cocktail were probed with anti-PepA, anti-PepB and anti-PepC antibodies. The light grey box denotes lanes that contain proteins not relevant to this study.

Supplementary Fig. 1

A

```
    5    10    15    20    25    30    35    40    45    50    55    60    65    70    75    80
MKLTK GCAYK YIIFT VLILA NILYD NKKRC MIKKN LRISS CGIIS RLLKS NSNYN SFNKN YNFTS AISEL QFSNF WNLDI

   85    90    95   100   105   110   115   120   125   130   135   140   145   150   155   160
LQKDI FSNIH NNKNK PQSYI IHKRL MSEKG DNNNN NHQNN NGNDN KKRLG SVVNN EENTC SDKRM KPFEE GHGIT QVDKM

  165   170   175   180   185   190   195   200   205   210   215   220   225   230   235   240
NNNSD HLQQN GVMNL NSNNV ENNNN NNSVV VKKNE PKIHY RKDYK PSGFI INNVT LNINI HDNET IVRSV LDMDI SKHNV

  245   250   255   260   265   270   275   280   285   290   295   300   305   310   315   320
GEDLV FDGVG LKINE ISINN KKLVE GEEYT YDNEF LTIFS KFVPK SKFAF SSEVI IHPET NYALT GLYKS KNIIV SQCEA

  325   330   335   340   345   350   355   360   365   370   375   380   385   390   395   400
TGFRR ITFFI DRPDM MAKYD VTVTA DKEKY PVLLS NGDKV NEFEI PGGRH GARFN DPHLK PCYLF AVVAG DLKHL SATYI

  405   410   415   420   425   430   435   440   445   450   455   460   465   470   475   480
TKYTK KKVEL YVFSE EKYVS KLQWA LECLK KSMAF DEDYF GLEYD LSRLN LVAVS DFNVG AMENK GLNIF NANSL LASKK

  485   490   495   500   505   510   515   520   525   530   535   540   545   550   555   560
NSIDF SYARI LTVVG HEYFH NYTGN RVTLR DWFQL TLKEG LTVHR ENLFS EEMTK TVTTR LSHVD LLRSV QFLED SSPLS

  565   570   575   580   585   590   595   600   605   610   615   620   625   630   635   640
HPIRP ESYVS MENFY TTTVY DKGSE VMRMY LTILG EEYYK KGFDI YIKKN DGNTA TCEDF NYAME QAYKM KKADN SANLN

  645   650   655   660   665   670   675   680   685   690   695   700   705   710   715   720
QYLLW FSQSG TPHVS FKYNY DAEKK QYSIH VNQYT KPDEN QKEKK PLFIP ISVGL INPEN GKEMI SQTTL ELTKE SDTFV

  725   730   735   740   745   750   755   760   765   770   775   780   785   790   795   800
FNNIA VKPIP SLFRG FSAPV YIEDN LTDEE RILLL KYDSD AFVRY NSCTN IYMKQ ILMNY NEFLK AKNEK LESFN LTPVN

  805   810   815   820   825   830   835   840   845   850   855   860   865   870   875   880
AQFID AIKYL LEDPH ADAGF KSYIV SLPQD RYIIN FVSNL DTDVL ADTKE YIYKQ IGDKL NDVYY KMFKS LEAKA DDLTY

  885   890   895   900   905   910   915   920   925   930   935   940   945   950   955   960
FNDES HVDFD QMNMR TLRNT LLSLL SKAQY PNILN EIIEH SKSPY PSNWL TSLSV SAYFD KYFEL YDKTY KLSKD DELLL

  965   970   975   980   985   990   995  1000  1005  1010  1015  1020  1025  1030  1035  1040
QEWLK TVSRS DRKDI YEILK KLENE VLKDS KNPND IRAVY LPFTN NLRRF HDISG KGYKL IAEVI TKTDK FNPMV ATQLC

 1045  1050  1055  1060  1065  1070  1075  1080  1085
EPFKL WNKLD TKRQE LMLNE MNTML QEPNI SNNLK EYLLR LTNKL
```

B

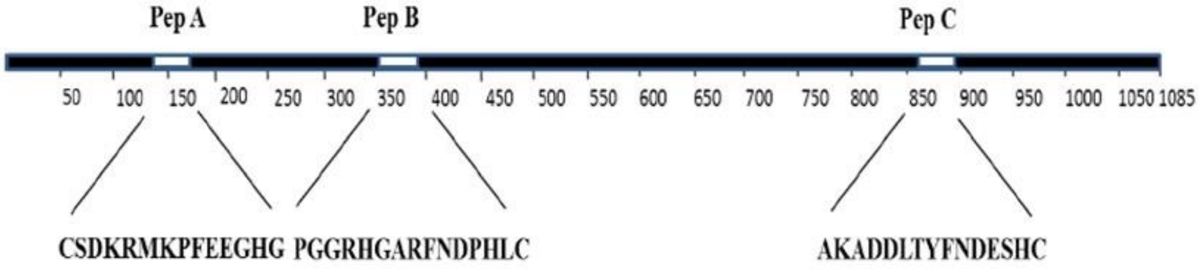

Supplementary Fig. 2

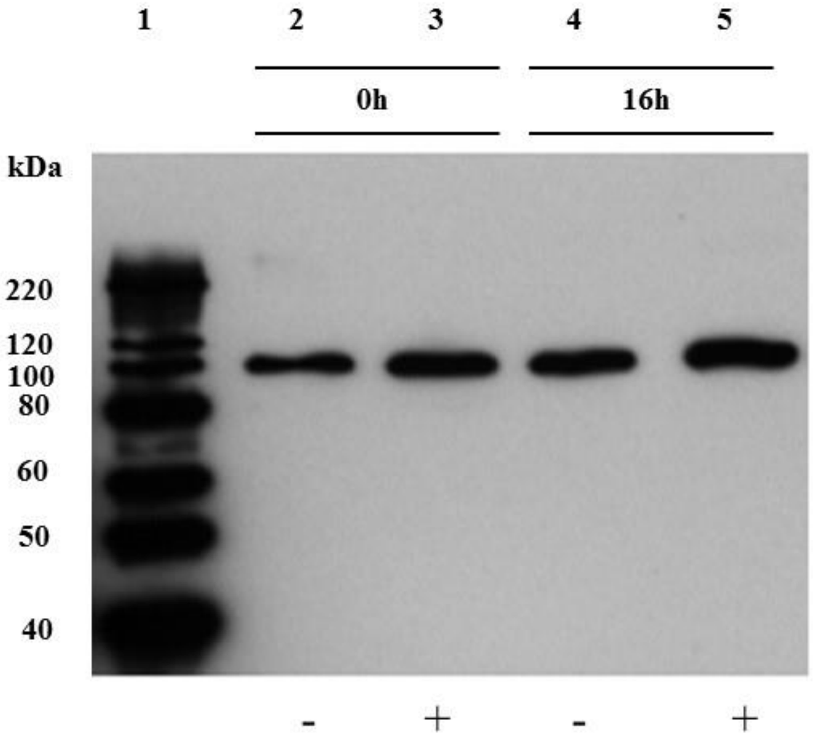

Supplementary Fig. 3

A. Cells probed with anti-*Pf*M1AAP antibodies

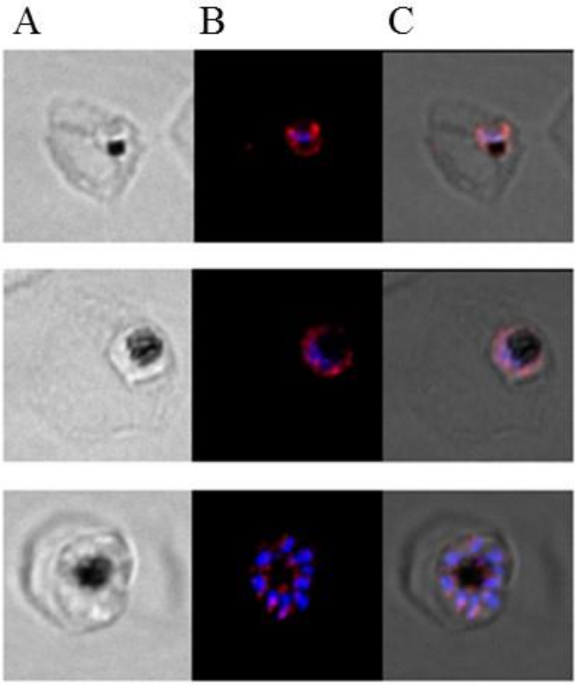

B. Cells probed with anti-*Pf*M17LAP antibodies

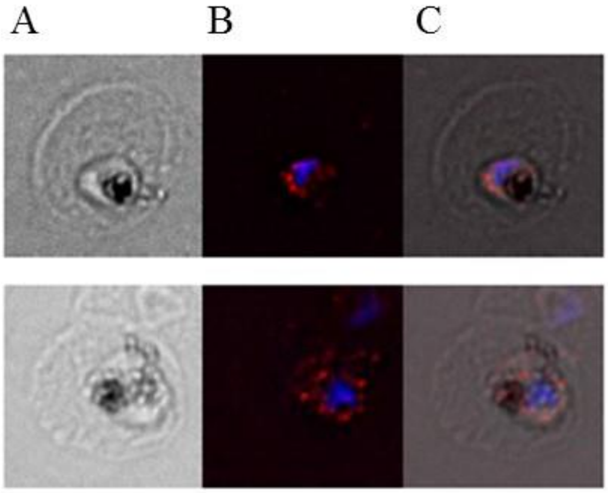

Supplementary Fig. 4

A. Cells probed with anti-*Pf*M1AAP antibodies

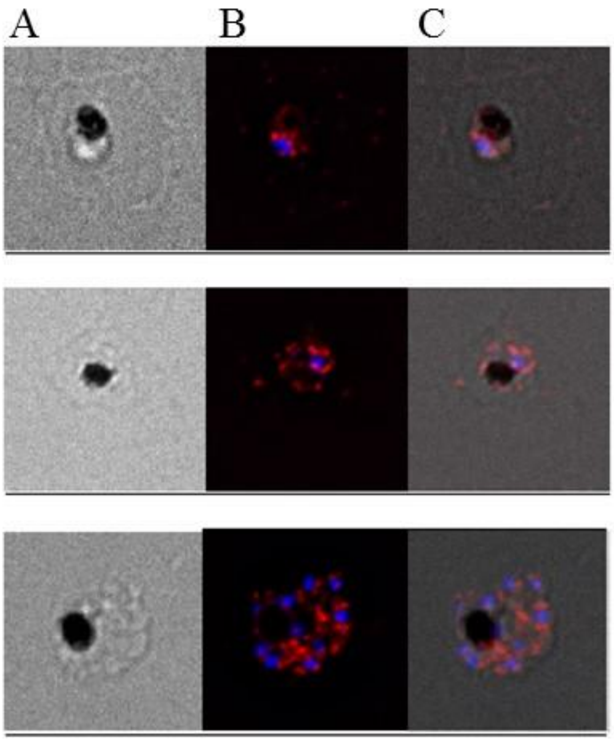

B. Cells probed with anti-*Pf*M17LAP antibodies

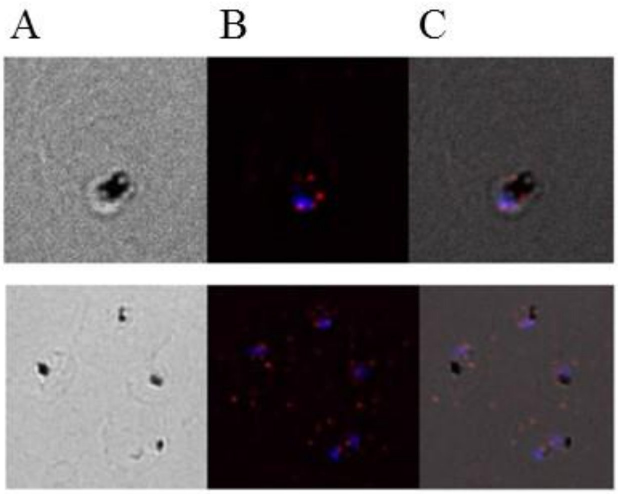

Supplementary Fig. 5

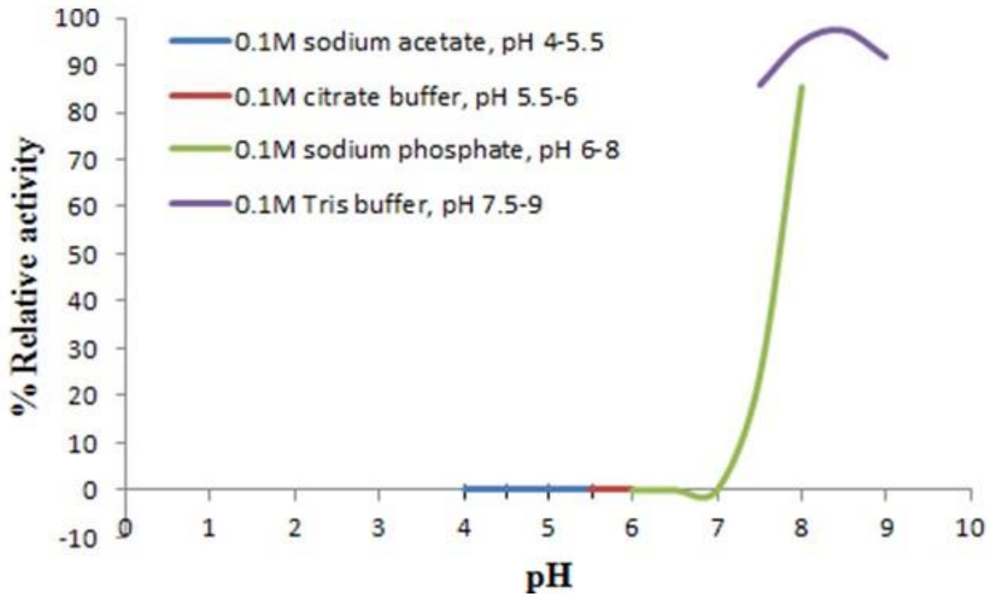

Supplementary Fig. 6

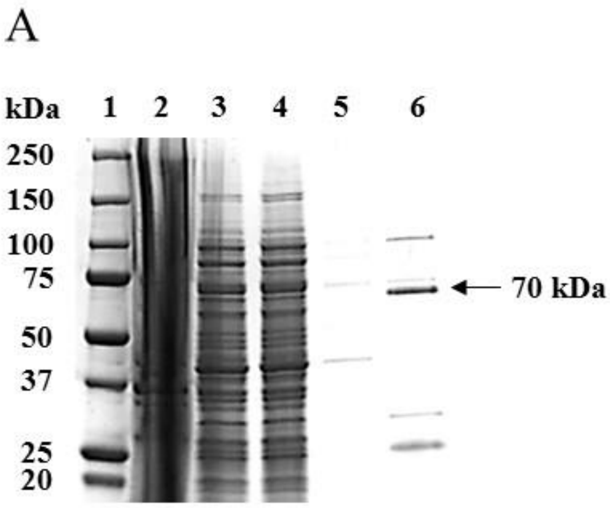

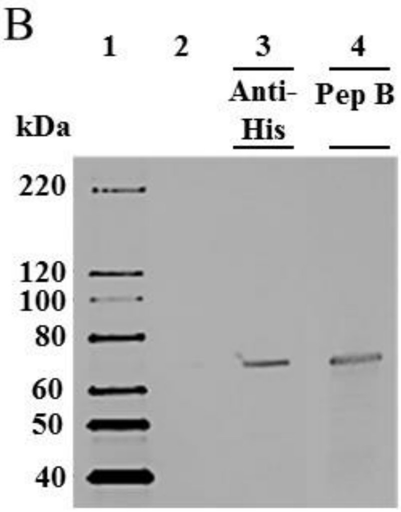

Supplementary Fig. 7

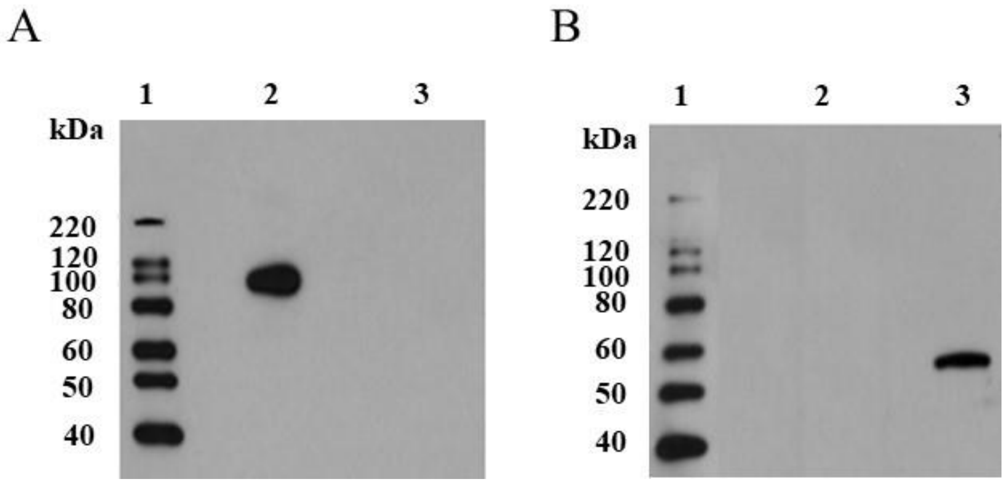

Supplementary Fig. 8

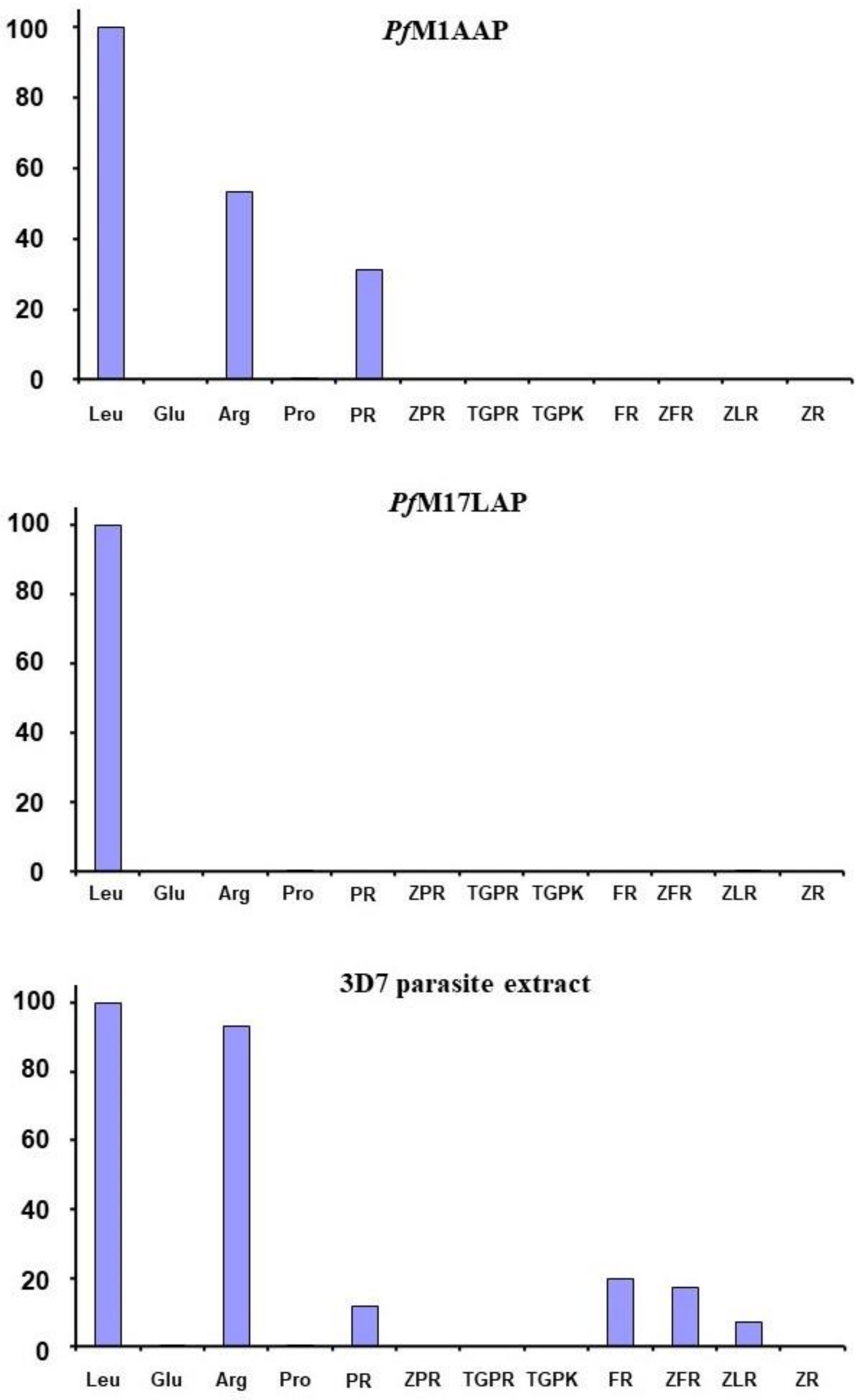

Supplementary Fig. 9A

anti-*Pf*M1AAP

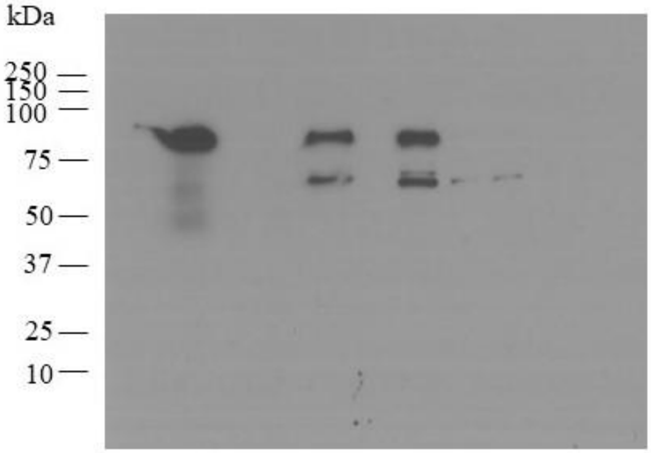

anti-*Pf*M17LAP

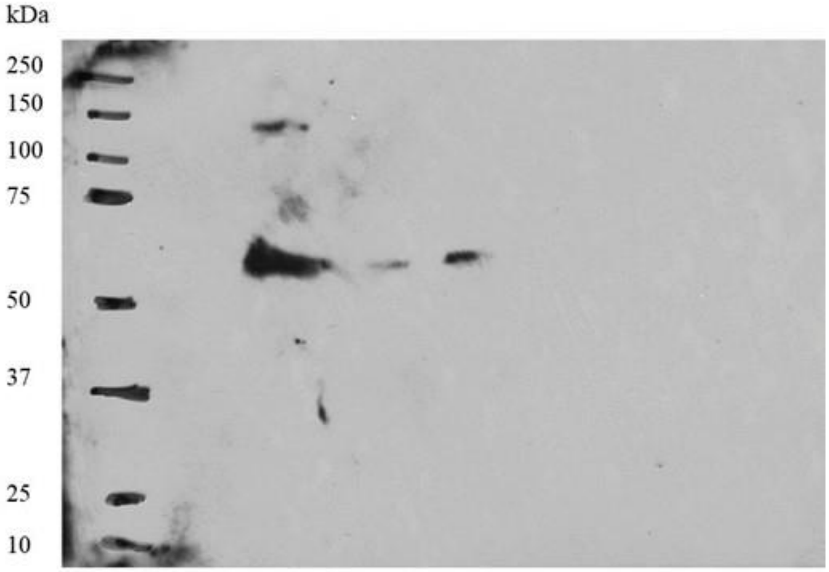

anti-plasmepsin 1

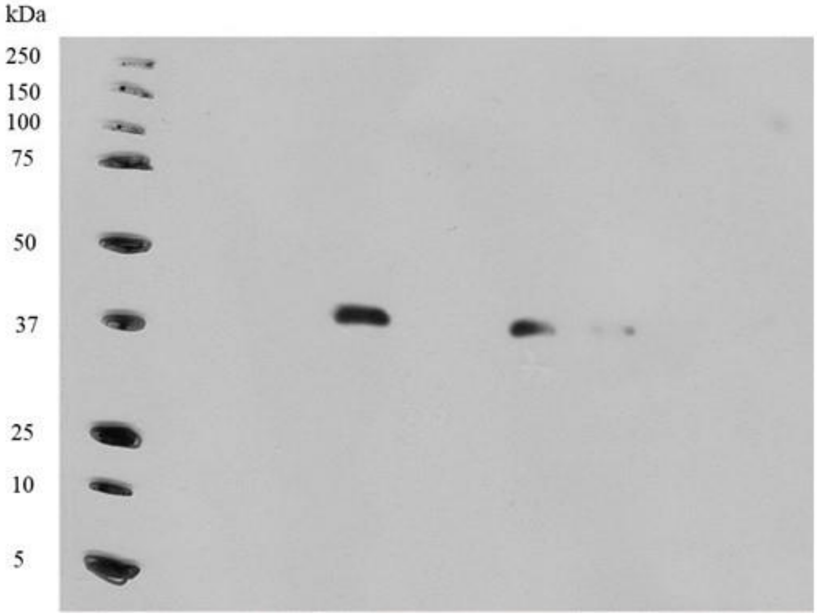

Supplementary Fig. 9B

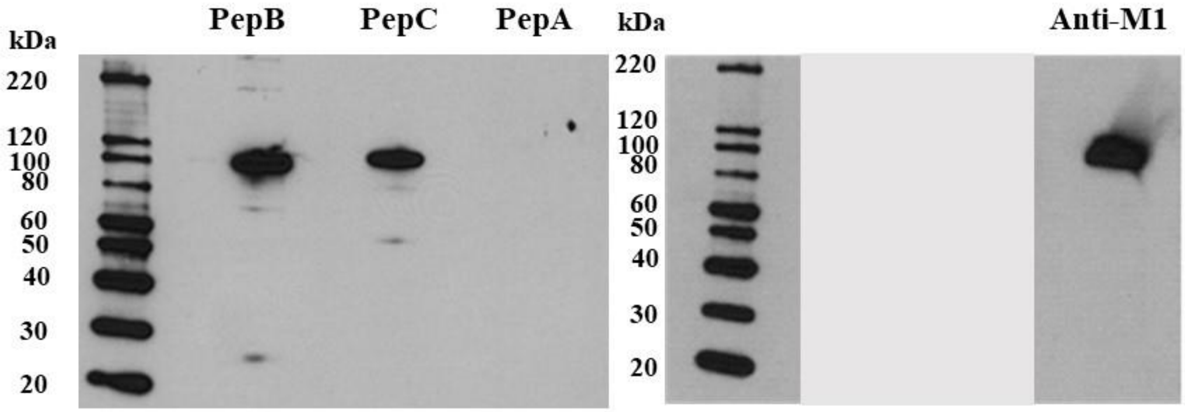

Supplementary Fig. 9C

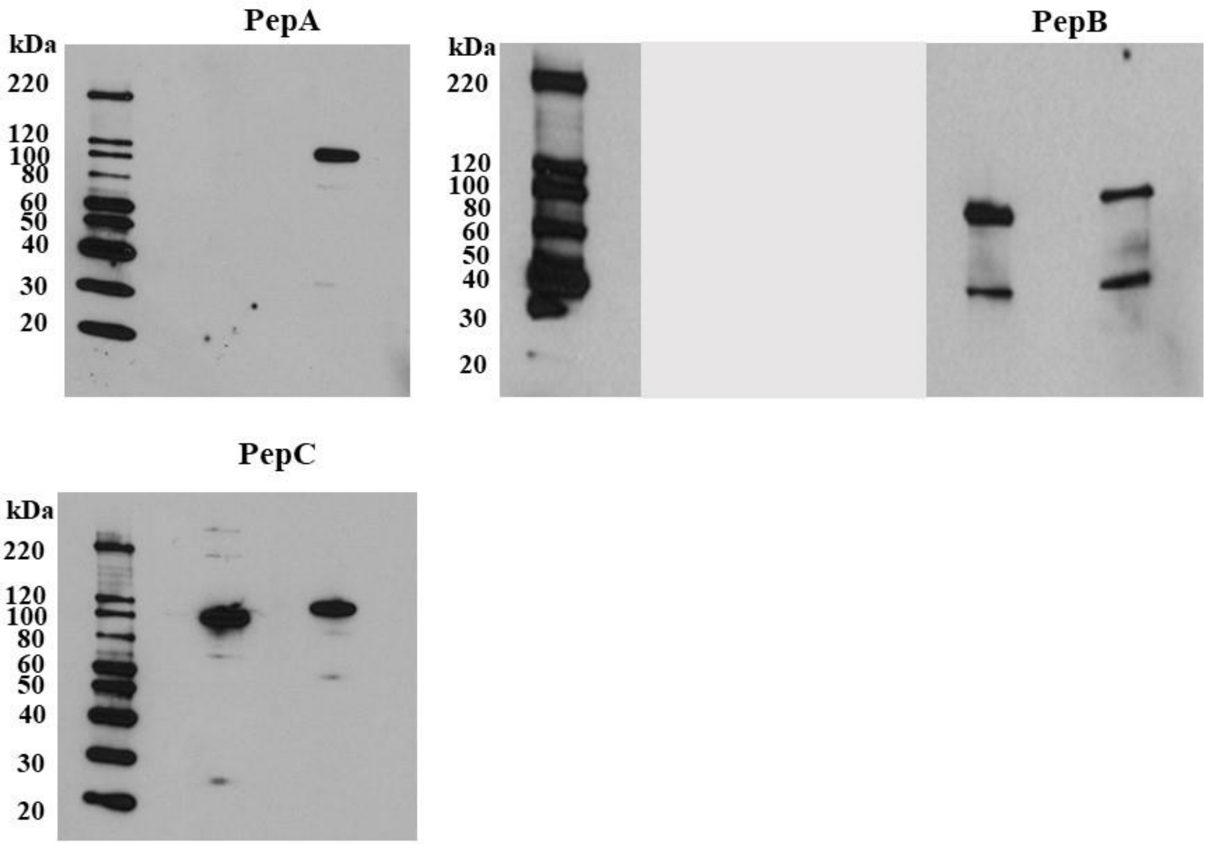

Supplement: Supplementary file 1 — Supplementary Figures. [file 41598_2021_82499_MOESM1_ESM.pdf]
